# Recent Advancements and Strategies for Overcoming the Blood–Brain Barrier Using Albumin-Based Drug Delivery Systems to Treat Brain Cancer, with a Focus on Glioblastoma

**DOI:** 10.3390/polym15193969

**Published:** 2023-10-02

**Authors:** Camelia-Elena Tincu (Iurciuc), Călin Vasile Andrițoiu, Marcel Popa, Lăcrămioara Ochiuz

**Affiliations:** 1 Department of Natural and Synthetic Polymers, “Cristofor Simionescu” Faculty of Chemical Engineering and Protection of the Environment, “Gheorghe Asachi” Technical University, 73, Prof. Dimitrie Mangeron Street, 700050 Iasi, Romania; camelia_tincu83@yahoo.com; 2 Department of Pharmaceutical Technology, Faculty of Pharmacy, “Grigore T. Popa” University of Medicine and Pharmacy, 16, University Street, 700115 Iasi, Romania; ochiuzd@yahoo.com; 3 Apitherapy Medical Center, Balanesti, Nr. 336-337, 217036 Gorj, Romania; dr_calin_andritoiu@yahoo.com; 4 Specialization of Nutrition and Dietetics, Faculty of Pharmacy, Vasile Goldis Western University of Arad, Liviu Rebreanu Street, 86, 310045 Arad, Romania; 5 Faculty of Dental Medicine, “Apollonia” University of Iasi, 11, Pacurari Street, 700511 Iasi, Romania; 6 Academy of Romanian Scientists, 3 Ilfov Street, 050045 Bucharest, Romania

**Keywords:** glioblastoma, albumin nanoparticles, overcoming blood–brain barrier, functionalization of nanoparticles

## Abstract

Glioblastoma multiforme (GBM) is a highly aggressive malignant tumor, and the most prevalent primary malignant tumor affecting the brain and central nervous system. Recent research indicates that the genetic profile of GBM makes it resistant to drugs and radiation. However, the main obstacle in treating GBM is transporting drugs through the blood–brain barrier (BBB). Albumin is a versatile biomaterial for the synthesis of nanoparticles. The efficiency of albumin-based delivery systems is determined by their ability to improve tumor targeting and accumulation. In this review, we will discuss the prevalence of human glioblastoma and the currently adopted treatment, as well as the structure and some essential functions of the BBB, to transport drugs through this barrier. We will also mention some aspects related to the blood–tumor brain barrier (BTBB) that lead to poor treatment efficacy. The properties and structure of serum albumin were highlighted, such as its role in targeting brain tumors, as well as the progress made until now regarding the techniques for obtaining albumin nanoparticles and their functionalization, in order to overcome the BBB and treat cancer, especially human glioblastoma. The albumin drug delivery nanosystems mentioned in this paper have improved properties and can overcome the BBB to target brain tumors.

## 1. Introduction

Glioblastoma multiforme (GBM) is a highly aggressive form of cancer and the most prevalent primary malignant tumor found in the brain and central nervous system (CNS) [1]. On average, patients with GBM have a low median overall survival rate of only 15 months. GBM is in the higher grade (IV) of primary brain tumors and is much more common in men. The incidence of GBM in recent years has been increasing. However, it is still challenging to determine the causes of its occurrence, and this is why additional research on the etiology and treatment of GBM tumors should continue. It should also be noted that the current therapy just slightly extends the life of patients, but cannot cure the cancer itself [2]. Many strategies have been adopted to develop effective drug delivery systems for the brain. The main mechanisms by which drugs cross the blood–brain barrier (BBB) are absorption-mediated transcytosis, transporter-mediated transcytosis, and receptor-mediated endocytosis. The blood–brain tumor barrier (BBTB), similar to the BBB, is located between brain tumor tissues and microvessels formed by specialized endothelial cells, limiting the release of hydrophilic molecules into the tumor tissue. Proposed strategies for targeting the BBTB are mainly based on receptors expressed on tumors, such as epidermal growth factor receptors and integrin [3]. Nutrient transport molecules have attracted special attention for possible applications in targeted drug delivery using various carriers [4]. The effectiveness of drugs in overcoming the BBB is influenced by multiple factors, including the physical and chemical characteristics of the drugs, their ability to bind with proteins, cerebral blood flow, their clearance from the body, and the integrity of the BBB. First, the physicochemical properties, such as lipophilicity, hydrogen bonding formation, particle size, and surface charge, influence the permeability of the drug molecules through the BBB [5].

Temozolomide (TMZ) is the standard chemotherapy for the treatment of GBM and is either used alone or in combination with radiotherapy for the treatment of GBM. Studies show that it provides clinical benefits in patient survival [6]. TMZ has indiscriminately attacked DNA, and has been shown to cause damage in a patient’s hematopoietic stem cells, leading to dose-dependent hematological toxicity. TMZ is poorly soluble under physiological conditions and undergoes rapid hydrolysis that limits its antitumor efficacy (the TMZ half-life is 1.8 h and requires frequent administration) [7,8,9]. A prolonged therapy leads to the body’s resistance to TMZ and a poor reaction of the body in subsequent treatments, leading to a tumor recurrence in 60–75% of cases. Current research shows that the genetic profile of GBM leads to resistance to TMZ and radiation, but drug delivery across the BBB is a significant struggle in treating GBM [10].

The limitations of chemotherapy highlight the need for a delivery system to increase the drug’s therapeutic index. Several drug delivery systems (liposomes, solid lipid nanoparticles, nanocapsules, and polymer nanoparticles) were tested to highlight their effectiveness. However, the success of these formulations was limited due to the lack of a specific delivery into cancer cells. The drug delivery system for glioma therapy should target the tumor and have the ability to overcome the BBB [11]. Drugs encapsulated in various supports can improve tumor cell targeting via the diffusion of drugs across the BBB using different mechanisms, such as specific tumor-targeting mechanisms based on an enhanced permeability and retention (EPR) effect, with targeting molecules attached to delivery systems that bind to the tumor cell receptors and the diffusion of these nanosystems with incorporated drugs within the tumor ensuring a homogeneous distribution of anticancer drugs inside the tumor [12].

There are many types of biomolecules used for controlled and targeted drug delivery.

A method that has been developed to overcome the limitations of polymer nanoparticles is the use of lipid nanoparticles. These nanoparticles are advantageous as they have low production costs and do not involve the use of solvents in their preparation stage, which can cause high toxicity [13]. Liposomes were the first model of lipid-based nanoparticles. The FDA has approved Doxil^®^/Caelyx^®^, a PEGylated doxorubicin liposomal formulation for cancer treatment. Despite liposomes’ unique advantages, such as high biocompatibility, low toxicity, non-immunogenicity, and biodegradability, their applications have been limited due to some associated disadvantages. Phospholipids in liposomes can undergo oxidation and hydrolysis reactions, resulting in poor stability, a short shelf life, low encapsulation efficiency, and high production costs [14]. Solid lipid nanoparticles (SLNs) and nanostructured lipid carriers (NLCs) are innovative drug delivery systems that are designed to replace traditional delivery systems like polymeric nanoparticles, liposomes, and emulsions. These newer systems have numerous advantages, including protecting drugs from environmental factors and their potential for large-scale production using high-pressure homogenization techniques. They are biocompatible and biodegradable, exhibiting superior stability and release profiles compared to liposomes. Furthermore, they are considered safer than polymeric nanoparticles as they do not use organic solvents [15,16,17,18]. Solid lipid nanoparticles (SLNs) and nanostructured lipid carriers (NLCs) can help deliver drugs to target cells through various mechanisms, including passive and active targeting. In passive targeting, SLNs and NLCs utilize specific properties of the tumor microenvironment to enhance drug release based on the EPR effect. The surface of SLNs and NLCs was modified for active targeting to be recognized by overexpressed transporters or receptors in target cells, leading to selective targeting and reducing side effects [17]. Although these nanoparticles have numerous benefits, SLNs have drawbacks, like unexpected gelling, low encapsulation efficiency, and drug expulsion due to solid lipid recrystallization during storage. This makes it challenging to keep the drug encapsulated. In order to overcome these limitations, a liquid lipid was introduced into the SLN formulation, leading to the development of NLCs [19]. Another disadvantage is the burst effect caused by erosion that usually occurs with these formulations, which can cause toxicity in the body due to the drug dose being too high [15]. 

Drug delivery to the brain can be improved by using lipid nanoparticles. These nanoparticles can increase the drug’s retention time in the blood of the cerebral capillaries and induce a certain concentration of the drug from the blood to the brain tissues. This helps to overcome the BBB by opening tight junctions (TJs) and utilizing the transcytosis of the drug-loaded lipid nanoparticles through the endothelium layer. For optimal outcomes, the nanoparticles can be coated with polysorbate 80. Furthermore, lipid nanoparticles with a positive surface charge can enhance drug accumulation in the brain. One of the main drawbacks of using lipid nanoparticles for cerebral administration is that they can be detected by the reticuloendothelial system (RES) cells, which can lead to the rapid elimination of drug-loaded SLNs from the systemic circulation. The intravenous administration of these delivery systems also has a few disadvantages. Firstly, a significant amount of the drug is initially expelled due to matrix erosion, which can cause side effects. Additionally, limited clinical studies are available, and the capacity for encapsulating hydrophilic drugs is reduced. Furthermore, the drug may be lost before it reaches the target site in the body, and in the case of the systemic administration of cytotoxic drugs, there is a chance that the RES may eliminate them. Lastly, the accumulation of lipids in the liver and spleen could cause pathological changes [20]. In order to improve drug delivery systems for the treatment of brain cancer and administration to the CNS, researchers have explored new approaches. One such promising approach is the use of albumin-based nanoparticles, which can address the limitations of SNLs. In the upcoming paragraphs, we will summarize the role of albumin in the body and its benefits as a drug delivery system for brain delivery.

Serum albumin, a globular protein secreted within the body, has some advantages that have attracted the attention of researchers. Albumin is not toxic, is non-immunogenic, ensures excellent biocompatibility with the nanoparticles, and has high stability in water and diluted salt solutions. The half-life of albumin in the bloodstream is 19 days, so the drugs encapsulated in albumin-based nanoparticles can be maintained in the bloodstream for an extended period of time compared with free drugs due to their role in the body, which is to interact with lipophilic molecules, such as hormones, fatty acids, vitamins (C, D, folic acid), and minerals (copper, zinc, calcium). Albumin stabilizes the blood pH and is responsible for 80% of the osmotic pressure of plasma [21,22]. 

Research carried out until now has shown that various drugs, genes, peptides, vaccines, and antibodies can effectively bind to albumin. This protein can be successfully used to obtain delivery systems with a controlled and targeted release of the encapsulated bioactive compounds. Albumin-based delivery systems have a high drug-loading capacity, good biocompatibility, and biodegradability. Albumin-based nanoparticles are efficient, easy to prepare, have a well-defined and controllable size and shape, and have characteristics for surface modification. Albumin nanoparticles are dried via freeze-drying and could be successfully used in nanomedicine. These nanoparticles can be redispersed in injectable solutions, ensuring the stability of the encapsulated active biological compound and avoiding premature drug release, agglomeration, and precipitate formation [23]. 

Albumin has been frequently identified in the protein corona on the surface of different types of nanoparticles, thus modulating their tissue localization and cellular targeting. Large amounts of albumin are found in human blood (30–50 g/L), and albumin-based nanoparticles are considered excellent delivery systems due to their non-toxicity and non-immunogenicity. Albumin contains three functional groups—COOH, NH_2_, and SH—which determine an easy functionalization of the protein with different ligands, and the targeted release of the encapsulated drugs is improved. It was shown that the incubation of polystyrene-based microparticles/nanospheres with HSA reduced phagocytosis in the dendritic cells, even in the presence of opsonins such as immunoglobulins and human serum glycoproteins [24]. 

Albumin influences various delivery systems’ stability, pharmacokinetics, and biodistribution by binding to their surface and forming the protein corona. The development of albumin-based drug delivery systems with controlled and targeted drug delivery is gaining increased importance in cancer therapy. Magnetic nanoparticles can be directed to a specific area in the body using a magnetic field. This method can potentially target drug delivery in cancer treatment and can be enhanced with heat treatment and MRI monitoring [22,25]. Magnetite, Fe_3_O_4_, has perspectives in this area but requires surface functionalization to prevent aggregation. Protein-coating magnetic nanoparticles, such as with albumin, provides them with biocompatibility, biodegradability, lower immunogenicity, and low cytotoxicity, and increases the targeting efficiency in various tissues and cells [25].

Radiopharmaceuticals for diagnosis are used in subtherapeutic amounts and have an excellent safety profile. The most common radionuclide is the gamma technetium-99m (^99m^Tc) emitter, with a half-life of 9 h. Albumin can cover radionuclides used in imaging techniques to prevent their possible side effects by forming albumin nanocolloids. A human serum albumin nanocolloid labeled with the radionuclide ^99m^Tc was initially developed for magnetic resonance imaging in the diagnosis of inflammation but quickly became used in the field of lymphoscintigraphy. These albumin-based nanocolloids are recommended in most European guidelines as a procedure for sentinel node localization [26].

The efficiency of albumin-based delivery systems is determined by their ability to improve tumor targeting and accumulation. For example, the increased accumulation of albumin nanoparticles in tumors is due to the increased passive uptake mediated by the EPR effect. In addition, albumin can bind to special receptors expressed in cancer cells and improve the binding and internalization of the nanoparticles. Various tumors overexpress the 60 kDa glycoprotein receptor (gp60) and the secreted protein acidic and rich in cysteine (SPARC) [27,28].

The binding ability of the gp60, gp30, gp18, and FcRn receptors to albumin ensures the transcytosis of albumin-based nanoparticles within the tumor cells. Its accumulation in tumors is facilitated by interactions with the SPARC receptor and the EPR effect [25].

This paper is a literature review covering several aspects of the prevalence of tumoral brain cancer, especially human glioblastoma, and the currently adopted treatment. Also, this literature review presents some essential BBB features for drug delivery systems’ transport and some factors related to the blood–tumor–brain barrier that determine the poor effectiveness of drugs in brain tumor tissue. The properties and structure of serum albumin and its role in targeting brain tumors were presented. We have chosen to discuss serum albumins and albumin-based nanoparticles because they have been mentioned as having suitable properties and an essential role in cancer targeting, especially in brain cancer. Many papers have demonstrated that serum albumins could specifically bind to 60 kDa glycoprotein (gp60) and SPARC (an acidic and cysteine-rich protein), determining its uptake into cancer cells via transcytosis. Also, albumin-based delivery systems have the ability to avoid the efflux mechanisms of the drug, determining an improved absorption of albumin-based nanoparticles into brain tumors. The novelty of this review article consists of a critical analysis of the progress made by researchers until now in developing albumin-based nanoparticles that can improve the treatment of brain cancer, especially glioblastoma multiform. This review article provides a new comprehensive analysis of albumin-based nanoparticles that have been used in research studies for the diagnosis and treatment of brain cancer. It also discusses the techniques for modifying and administering these nanoparticles to overcome the BBB, and the specific targeting methods used to treat malignant brain tumors. Several review articles have been published that detail the methods for producing albumin-based nanoparticles and their potential use in overcoming drug resistance for various types of cancer [23,27].

Additionally, another review article explores the application of serum albumin-based delivery systems as nanoprobes for cancer diagnosis and treatment [28]. However, they do not describe in detail the mechanisms underlying the treatment of a specific type of cancer and the biological limitations that appear in the administration of specific anticancer drugs. A recently published article discusses using albumin nanoparticles for administering chemotherapeutic drugs in breast cancer therapy, exploring various multifunctional theranostics [29]. In order to enhance the effectiveness of albumin-based nanoparticles that are used for treating different types of cancer, researchers must concentrate on comprehending and communicating the relationship between the preparation conditions and the intended therapeutic use. Scientists must take an interdisciplinary approach to develop multifunctional, next-generation albumin-based delivery systems to treat brain cancer.

Albumin represents a promising candidate for radiopharmaceuticals’ conjugation and for the magnetic nanoparticle coating that is used in the theranostic field, and can provide biocompatibility, prolonged blood circulation, immunogenicity, and low toxicity. The drug delivery nanosystems mentioned in this paper have improved properties and can overcome the BBB and target brain tumors.

Considering the benefits of using albumin as a drug delivery system and the fact that there are only two clinical trials (phase 1 and 2) for the treatment of glioblastoma using these delivery systems, we believe that this literature synthesis could help researchers in the field to develop new drug delivery systems based on albumin with improved properties to overcome the biological barriers that limit CNS drug delivery and aid in the early diagnosis and treatment of brain cancer.

## 2. Classification of Brain Tumors

The most common brain tumors (gliomas) arise from glial cells, ranging from low-infiltrating to highly aggressive forms. In 2007, the World Health Organization (WHO) classified gliomas into four categories based on their histopathological features. These features include the mitotic index, anaplasia, cytological atypia, microvascular proliferation, and necrosis: grade I (i.e., pilocytic astrocytoma), grade II (i.e., astrocytomas and oligodendrogliomas), grade III (i.e., anaplastic astrocytomas and oligodendrogliomas) and grade IV (i.e., glioblastoma multiforme). In 2016, the WHO included molecular diagnostic criteria for infiltrating gliomas, including isocitrate dehydrogenase mutation, chromosome 1p/19q deletion, and histone mutations in the classification [30]. However, malignant or high-grade (III and IV) gliomas are characterized by a poor prognosis. In addition, 8–10% of adult cancer patients develop brain metastases, with a considerably variable incidence between different types of primary cancer. Lung, breast, colon, kidney, or melanoma cancer can lead to brain metastases, of which 70% are from lung and breast cancer [31]. 

## 3. Prevalence and Treatment

It is well-known that glioblastoma (GBM) stands out as one of the most aggressive forms of cancer. It is a primary malignant tumor that affects the brain and central nervous system (CNS), accounting for 14.5% of all CNS tumors and 48.6% of all types of CNS cancer [1]. Unfortunately, patients diagnosed with GBM have a low median overall survival rate of only 15 months. This type of cancer originates from astrocytic glial cells [32] and is classified as a high-grade (grade IV) malignant glioma. It is not easy to establish the incidence of GBM because it varies according to different reports, from 3.19 cases per 100,000 people [33,34] to 4.17 per 100,000 people [2,35]. The incidence in the pediatric population is 0.85 per 100,000, where pediatric glioblastoma multiforme represents 3–15% of primary brain tumors [2,36,37,38] in this age group. It is important to note that the second most common form of cancer in children is primary central nervous system (CNS) tumors [2]. The incidence of GBM shows slight variation based on location and is most frequently found in the frontal, temporal, parietal, and occipital lobes. However, it can also affect other areas, such as brain stem cells, the cerebellum, and the spinal cord, although less commonly [39]. Age is a significant factor in the incidence of GBM, with almost half of all cases diagnosed in individuals aged 40–65 years [40,41]. In addition, GBM is slightly more prevalent in males than females, and in Caucasians compared to other ethnicities [42]. Treating GBM requires a comprehensive approach involving multiple disciplines. The most effective plan of action involves a thorough surgical procedure to remove as much of the tumor as possible, followed by radiotherapy and a concomitant oral administration of TMZ, an alkylating chemotherapeutic agent. After that, adjuvant chemotherapy with TMZ is given [43,44].

Removing GBM through surgery represents a significant challenge due to the invasive nature of tumors that typically occur in essential brain regions that control speech, motor function, and sensory perception. Unfortunately, surgery alone cannot completely eliminate the primary tumor mass, as infiltrated tumor cells remain in the surrounding brain tissue, leading to disease progression or recurrence [45]. Even with advances in surgical resection, the prognosis for patients with GBM remains poor, with a median survival of 15 months [41].

Despite maximal resection therapy and multiple treatment options, approximately 70% of patients with GBM will experience disease progression within one year after diagnosis [46], and less than 5% of patients survive five years after diagnosis [47].

Chemotherapy is an alternative treatment for this type of cancer. However, its effectiveness is limited by the toxic effects on healthy cells, the chemoresistance of tumor cells, and the poor selectivity of anticancer drugs. Finally, the BBB is the principal limit in releasing the chemotherapeutic agents into the tumor mass [48]. Thus, the chemotherapeutics currently used for high-grade glioma are still limited to a few chemical compounds with a limited administration of up to six months [49]. After surgery, the preferred first-line treatment for low-risk or progressive gliomas is oral TMZ. However, the Radiation Therapy Oncology Group recommends combining radiation therapy with chemotherapeutics such as procarbazine, lomustine, and vincristine as the standard treatment method [50]. The Food and Drug Administration (FDA) has recognized oral TMZ as the standard chemotherapy for GBM and anaplastic astrocytoma.

Bevacizumab is a monoclonal antibody that specifically binds to the vascular endothelial cell growth factor (VEGF). Despite the FDA’s accelerated approval of bevacizumab for brain tumors, based on its effectiveness against recurrent glioblastoma, this anti-angiogenic therapy failed to improve overall patient survival, although it effectively reduced or stopped tumor growth [51].

In 1996, the FDA approved biodegradable polyanhydride wafers loaded with carmustine (Gliadel^®^) for the chemotherapy of recurrent high-grade gliomas. Patients with recurrent tumors benefited from the 8-week survival increase when wafers were administered after the second surgery. Survival was increased by 2.3 months in patients with early-diagnosed tumors undergoing primary tumor resection followed by wafer administration [52].

Glioma oncogenesis is complicated, with different barriers preventing the drug from being delivered to the tumor site. The drugs must overcome three main barriers to treat brain tumors: the BBB, the blood–brain tumor barrier (BBTB), and the relatively low EPR effect. New strategies are required to develop a drug delivery system that is able to overcome these barriers and be directed to the tumor site [3].

## 4. The Blood–Brain Barrier (BBB)

The BBB, or blood–brain barrier, is a semipermeable physiological membrane that separates the brain tissue from the blood. It is a complex system consisting of endothelial cells, astroglia, pericytes, and perivascular mastocyte cells. Its primary function is maintaining the central nervous system’s homeostasis and preventing harmful substances, such as toxins and germs, from entering the brain [53].

The BBB consists of endothelial cells that are in close proximity to the basement membrane. Astrocytes act as a protective barrier between neurons and capillaries. They surround the cerebral capillaries and ensure that the permeability of the BBB is continuously regulated [54]. 

Several distinguishing features of the brain endothelium contribute to its unique barrier properties, which differ from those of peripheral tissues’ endothelium. 

The TJs found in the brain endothelium are highly complex and have been observed through imaging techniques as a chain-like network consisting of intramembranous particles that are extremely efficient in blocking intracellular clamps [55]. Researchers have established that transmembrane proteins, such as occludin and claudin, play a significant role in TJ structure and regulation [56]. They limit the paracellular flow of hydrophilic molecules across the BBB. Together with adherens junctions, they form a tight structure between adjacent endothelial cells, maintaining distinct tissue spaces by separating the luminal part from the abluminal one of the plasma membrane. In normal physiological conditions, molecules weighing more than 180 Da cannot overcome TJs [57]. These junctions significantly restrict even the mobility of small ions such as Na^+^ and Cl^−^; thereby, the transendothelial electrical resistance (TEER), which generally has values between 2 and 20 Ω·cm^2^ in peripheral capillaries, can reach the value of 1000 Ω·cm^2^ in the cerebral epithelium. However, the barrier properties of the TJs can vary between brain endothelial cells from different areas. 

As drug substances move from the capillaries of the endothelial cells to the postcapillary venules, their ability to permeate through TJs increases [58]. When designing drug delivery systems, most researchers focus on the BBB capillaries. However, postcapillary venules are a better option for transporting nanoparticles across the BBB because they are more easily accessible within the vascular segment [59]. 

Three essential transmembrane proteins govern the maintenance of TJs: claudins, occludin, and tight junction adhesion molecules (JAMs) [60].

Therapeutical substances can overcome the BBB through various mechanisms, including transmembrane diffusion, saturable transport, endocytosis, and extracellular pathways [61]. Overcoming the BBB requires lipid-soluble molecules that are smaller than 400 Da and not substrates of active efflux transporters. If a molecule does not meet these criteria, it can only cross the BBB through either carrier-mediated transport (CMT) or receptor-mediated transport (RMT) [62].

In some pathological conditions, such as inflammation, brain trauma, or ischemic vascular accidents, the BBB is compromised and allows for the passage of hydrophilic substances more easily. Because polysaccharides are large hydrophilic molecules, they are not expected to overcome the BBB under normal conditions unless they have been actively transported via CMT or RMT [63]. CMT is used for small hydrophilic nutrients such as glucose or amino acids [64]. RMT uses the vesicular trafficking mechanisms of the brain endothelial cells to deliver a range of proteins, including transferrin, insulin, leptin, and lipoproteins, to the CNS [65]. 

The existence of the BBB was first reported in 1885 by Elrich. After injecting a dye into the plasma, it was found in all organs except the brain and spinal cord. The first interpretation was that of the lack of the dye’s affinity to the CNS tissue. Later, the lack of a transfer of substances from the cerebrospinal fluid to the blood was also described. Lewandowski first introduced the term *blood–brain barrier* in 1900, and its structure began to be characterized in 1960, with subsequent additions [66].

Anatomically, the endothelial cells of the cerebral microvessels are distinguished from the rest of the cells of the same kind by a large number of mitochondria, the absence of fenestrations, low pinocytotic activity, and the presence of TJs [64]. Transmembrane proteins such as occludin and claudin-5, as well as junction adhesion molecules, are expressed in TJs. Zonula occludens (ZO) act as scaffolding proteins that connect these proteins to the cytoskeleton and help to maintain structural integrity and reduce permeability through the BBB [60,67,68,69]. The pericytes located on the outer surface of endothelial cells are irregularly attached to the basement membrane. The basement membrane is composed of various components, including type IV collagen fibers, proteoglycans, heparin sulfate, laminin, fibronectin, and other cell-matrix proteins [70]. The basement membrane continues with the astrocyte’s end feet, which cover the cerebral capillaries.

**Figure 1:**
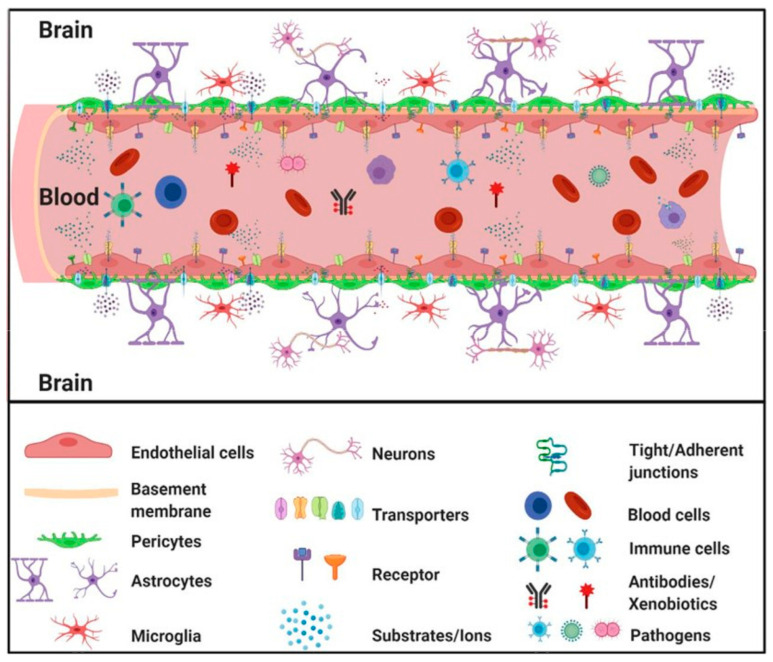
Schematic structure of the blood–brain barrier (BBB) with its constituent elements [80].

Astrocytes serve as mediators between neurons and cerebral microvessels, always maintaining the proper regulation of cerebral microcirculation [71,72]. In response to hypoxia or brain trauma, pericytes have the ability to move away from cerebral vessels, which can cause an increase in BBB permeability. Various pathological conditions can lead to BBB damage, including those associated with cerebral oxidative stress, such as ischemia, alcohol abuse, cocaine use, and neuroinflammation [73,74]. The inability of the BBB to function properly is a significant factor in the development and pathophysiology of a range of neurological diseases. These may include stroke, multiple sclerosis, brain trauma, neurodegenerative disorders, meningitis, epilepsy, optic neuromyelitis (Devic disease), trypanosomiasis (sleeping disease), progressive multifocal leukoencephalopathy, De Vivo disease, Alzheimer’s, and HIV encephalopathy. Disruption of the BBB can cause the dysregulation of the level of ions, the disturbance of signaling homeostasis, and the infiltration of immune cells and molecules into the CNS. These factors are likely to contribute to the dysfunction and degeneration of neurons [56,75]. Figure 1 schematically shows the structure of the BBB with its constituent elements.

The BBB serves multiple functions, such as providing essential nutrients to the brain and mediating the efflux of waste products. The interstitial fluid has a composition similar to blood plasma but contains lower levels of proteins and reduced amounts of K^+^ and Ca^2+^ ions. However, it has a higher concentration of Mg^2+^ ions. The BBB protects the brain against any changes in the levels of ions that may arise after eating or exercising. These changes have the potential to disrupt the signaling between neurons and axons [76,77,78]. The BBB plays an essential role in separating the neurotransmitters and neuroactive agents that act in the central nervous system, and those that act in the peripheral tissues and blood. The choroid plexus epithelium (the blood–cerebrospinal fluid barrier between the CSF and the extracellular space of the brain) that is responsible for cerebrospinal fluid production also contributes to this process and has other roles, such as the secretion of growth factors. The continuous flow and drainage of cerebrospinal and interstitial fluid further aid in the homeostasis of the brain microenvironment [79].

## 5. Other Central Nervous System Barriers

The cerebral ventricles and subarachnoid space contain the cerebrospinal fluid (CSF) secreted by the choroid plexuses into the lateral (third and fourth) ventricles. Three barrier layers (one being the BBB, as described previously) limit and regulate the molecular exchange at the interface between the blood and neuronal tissue or its fluid spaces [81,82].

The brain’s largest barrier is the BBB, which is formed by the cerebral endothelium. The barrier that separates the blood and cerebrospinal fluid (CSF) is made up of epithelial cells in the choroid plexus and vascular arachnoid epithelium around the brain, which together form the blood–CSF and CSF–blood barriers [83]. The central nervous system has additional interfaces that connect with the blood and neural tissue, namely the blood–retinal barrier and the blood–spinal cord barrier. These barriers perform crucial protective roles for the brain, protecting it against harmful pathogens and regulating its immunologic status [84]. 

The blood–cerebrospinal fluid barrier (BCSFB) is a protective barrier that separates the blood from the CSF. It is formed from the epithelial cells of the choroid plexus that regulate the entry of substances into the brain’s ventricles [85]. The BBB capillaries’ endothelium ensures the reverse flow of the brain’s extracellular fluid [86]. 

The arachnoid barrier consists of a vascular arachnoid epithelium [87]. It contributes insignificantly to blood–brain exchange due to its limited surface area compared to other barriers [88]. Individual neurons are rarely larger than 8–20 μm from a brain capillary, although the size can be on the order of millimeters in a given CSF compartment. The barrier controls the microenvironment regulation close to the brain cells [89,90]. 

The presence of intracellular and extracellular enzymes creates a “metabolic barrier”. Ectoenzymes like peptidases and nucleosidases can metabolize enzymes and ATP (Adenosine 5′-triphosphate), while intracellular enzymes like monoamine oxidase and cytochrome P450 can inactivate various neuroactive and toxic substances [91].

## 6. Transport through the BBB

The main BBB functions are to restrict the entry of unwanted substances in the brain that circulate into the blood to prevent the loss of necessary substances, and, at the same time, to provide the means for the transport of O_2_, CO_2_, and glucose to support the metabolic requirements of the brain cells. The essential substances transferred inside or outside of the brain parenchyma are water, glucose, O_2_, CO_2_, and, in smaller amounts, amino acids. Most fluxes of these substances must overcome the BBB because blood flow to the choroid plexuses is insufficient to provide or remove the required amounts [92]. 

**Figure 2:**
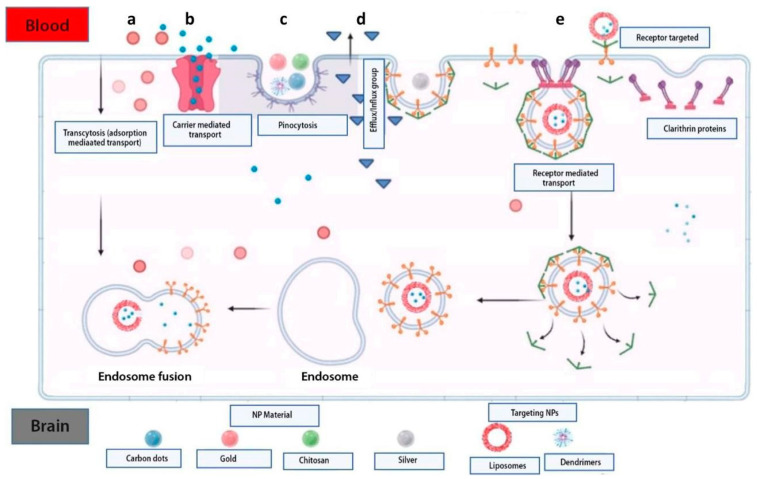
Methods used to overcome the BBB via controlled drug release systems. Nanoparticles based on biopolymers are transported via the cellular adsorption method by electrostatic forces using surface charges (**a**). Administration of small molecules of active substances encapsulated in nanoparticles using transport mediated by membrane proteins (**b**). Transport via endocytosis of natural inorganic nanoparticles into the cell (**c**). Mechanism of the efflux pump that causes drug resistance in the brain (**d**). Nanoparticle transport using surface receptors such as transferrin and LDL targeting receptors (**e**) [93].

Therefore, BBB has two main functions: protecting the brain and transporting substances [76]. Substances enter into the brain through two different pathways: paracellular and transcellular transport. TJs prevent molecules from passing through the paracellular pathway, while the transcellular pathway allows molecules to pass based on their electrochemical gradients, such as their concentration, electrical charge, and lipophilicity. Active transport, which requires adenosine triphosphate (ATP) as an energy source, drives molecules through the BBB against their concentration gradient. Other processes, such as pericyte and endothelial ion transporters, facilitate the movement of less lipophilic molecules. Additionally, endothelial transporters for various substances like carbohydrates, amino acids, monocarboxylates, hormones, fatty acids, nucleotides, organic anions, and cation transporters play a vital role in this process. Active endothelial efflux (ATP-binding cassettes) and receptor-mediated transporters also help to move substances through the BBB (Figure 2) [93]. 

The endothelial transporters of the brain that supply it with nutrients include the glucose transporter, several amino acid transporters (LAT1-system L for large neutral amino acids, y+), and the transporter for nucleosides, nucleobases, and many other substances [56,94]. About 90–95% [92,95] of the metabolism of 0.6 moles of glucose per day in the brain shows complete oxidation, consuming about 3.3 moles per day of O_2_ and producing the same amounts of CO_2_ and water daily [96].

During neuronal activity, more O_2_ enters into the brain parenchyma to provide the necessary means to increase the metabolism [97,98]. The O_2_ in the blood comes into contact with the hemoglobin from the red blood cells, and normal blood flow is adequate to support the activity of the O_2_ in the brain [99]. If there are relatively significant changes in the plasma glucose concentration, they have no effect on neurovascular coupling [100]. Conversely, even some minor increases in CO_2_ in the arterial blood or decreases in the CSF pH can cause vasodilation, and decreases in CO_2_ or increases in pH can produce vasoconstriction [101,102] and clinical consequences [103]. 

The brain’s glucose requirement is approximately 0.6 mol/day, with this glucose amount being able to exceed the BBB. The blood flow to the brain is, on average, 800 mL/min and contains about 400 mL/min of plasma, in which 5 mmol/l glucose is found, corresponding to about 20% of the required glucose. The experimentally measured amount of glucose in physiological plasma varies between 15 and 35%. *D*-glucose transport is very fast, while L-glucose is slow, comparable to other polar solutes such as sucrose and mannitol [104,105]. Glucose transport across the BBB is passive but mediated by specific transporters, such as GLUT1, expressed in endothelial cells’ luminal and abluminal membranes [106]. The glucose influx from the blood into the brain exceeds the glucose efflux from the brain into the blood, leaving behind a relatively large net flow of about 30% of the total amount that crosses the BBB [107]. After crossing the endothelial cells, glucose can be infiltrated into the astrocytes’ feet and does not diffuse through the space between them. A part of the glucose can be transported back from the astrocytes’ feet and through the endothelial cells, contributing to the efflux. 

The difference in the glucose concentration between the blood and the interstitial fluid causes a flow through the endothelial cells located in the basement membrane. The glucose present in the basement membrane needs to be transported to the rest of the brain. A certain amount of glucose in astrocytes is metabolized into lactic acid and is not directly transmitted to neurons. The supply of glucose to the brain increases during sustained neuronal activity [108,109,110]. 

It is essential to mention amino acid transport because the efflux of these molecules from the brain into the endothelial cells is connected to the transportation of Na^+^ ions. The importance of the functional polarity of the BBB has been demonstrated for the transport of amino acids [111]. The BBB greatly restricts amino acid influx, including the glutamate and glycine neurotransmitters, but allows a rapid, passive efflux of many other essential amino acids [112]. The BBB controls amino acid concentrations in the brain for their transport from the plasma to the cerebrospinal fluid and their active removal from the brain via Na^+^-dependent transporters located in the abluminal membrane (LAT1 and γ+) [113]. Five active transporters that require Na^+^ ions are present in the abluminal membrane and are responsible for the efflux of amino acids from the brain into the endothelial cells, and then into the blood [114]. Na^+^ is transported through the apical membrane of the epithelial cells, primarily via the Na^+^ ion pump, and through the basolateral membrane via cotransport with HCO_3_^−^. Cl^−^ is transported through the basolateral membrane via a Cl^−^/HCO_3_^−^ exchange, and through the apical membrane via multiple mechanisms. The transcellular transport of K^+^ ions is in the direction from the cerebrospinal fluid to the blood because its only transport route is through the basolateral membrane, which represents the cotransport of K^+^ and Cl^−^, which mediates the efflux from the epithelial cells [92].

The BBB is responsible for ionic homeostasis in the brain microenvironment. Through ion channels, the BBB can regulate the concentration levels of not only potassium ions (K^+^), but also of calcium (Ca^2+^) and magnesium (Mg^2+^) ions. The concentration of K^+^ in the blood plasma is about 1.8 times higher compared to the cerebrospinal and interstitial liquid [115,116]. Homeostatic regulation through ion channels (K+, Ca^2+^, and Mg^2+^) ensures the normal function of the neural network [88,117].

High-molecular-weight hydrophilic molecules such as peptides and proteins are generally excluded if they cannot be transported or mediated by a specific receptor or via less-specific adsorption-mediated transcytosis. However, the cerebral endothelium has a lower degree of endocytosis/transcytosis activity than the peripheral endothelium and determines transport activity through the BBB. Therefore, the term BBB covers a number of passive and active features of the brain [62,118,119].

Because TJs severely restrict the entry of hydrophilic drugs into the brain and there is a limited diffusion of large molecules such as peptides, as well as drug transport and delivery strategies to the CNS, these features must be considered [90]. The transendothelial electrical resistance (TEER) of cerebral microvessels is 100 to 500 times higher than that of non-cerebral capillaries [120]. However, the BBB is not a static barrier; there is a massive exchange of substances across the BBB through nutrient transport molecules. Nutrient transport molecules have attracted particular attention for possible applications in targeted drug delivery [120].

## 7. Blood–Brain Tumor Barrier

The oncogenesis of gliomas is complicated, with different barriers preventing drugs from reaching the tumor site. There are three main barriers to treating brain tumors: the BBB, the blood–brain tumor barrier (BBTB), and the relatively weak EPR effect [3]. 

The BBTB is similar to the BBB and serves as a barrier between brain tumor tissues and the microvessels composed of specialized endothelial cells. Its function is to limit the influx of hydrophilic molecules into the tumor tissue. As brain tumors advance, they infiltrate the surrounding healthy brain tissue. The BBB is damaged, and the BBTB is formed when the tumor cell clusters reach a specific size. Solid malignant tumors found in the peripheral tissues generally have a more permeable blood–tumor barrier than those growing in the brain. Over time, the BBTB becomes the main obstacle to nanosystems designed for drug delivery. The BBB is compromised in the case of malignant gliomas, and the permeability differs from the other regions. Gliomas that infiltrate around the tumors’ edge continue to utilize the available cerebral vasculature, but the BBB continues to restrict the delivery of specific chemotherapy drugs to the tumor.

**Figure 3:**
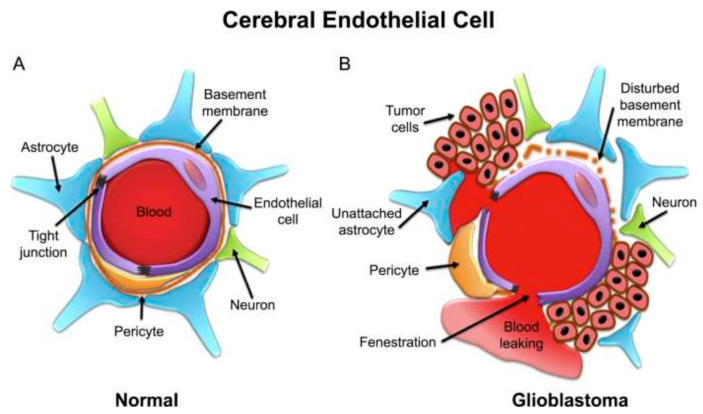
Schematic representation of cerebral capillary showing an endothelial cell in (**A**) normal and (**B**) glioblastoma conditions [122].

Identifying receptors in the BBB/BBTB offers a promising opportunity for the targeted administration of medications in glioma therapy. When targeting the BBTB, common strategies include focusing on receptors that are abundant in tumors, such as epidermal growth factor receptors and integrins. In order to avoid the BBB, methods such as opening TJs using a hyperosmotic mannitol solution or a chemical (such as bradykinin), blocking efflux drug transporters, and using receptor-mediated drug delivery systems can be employed to enhance the selective release of drugs into brain tumors. Additionally, peptide-coated nanoparticles that increase cell permeability may effectively overcome the BBTB [75,121]. Figure 3 schematically shows the cerebral capillary, showing the normal endothelial cell and in glioblastoma.

## 8. Factors Influencing the Passage of Drug Molecules through the BBB

Several drug delivery methods have been developed to overcome the barriers that limit the delivery of drugs or potential therapeutic agents in the CNS. These strategies can be categorized into invasive, non-invasive, and miscellaneous techniques [123,124,125].

Drugs’ ability to overcome the BBB depends on several factors, such as a drug’s physicochemical properties, protein binding, the cerebral blood flow, drug clearance, and barrier integrity. The permeability of drug molecules through the BBB is influenced by its physicochemical features, such as the lipophilicity, the ability to form hydrogen bonds, the particle size, and the influence of the surface charge. A drug must possess both lipophilic and hydrophilic characteristics to reach its intended target in the brain effectively. If a drug is highly lipophilic, it risks being trapped within the cell membrane, which reduces its ability to reach the brain parenchyma. On the other hand, highly hydrophilic drugs cannot cross the lipid-soluble cell membrane, which means that the partition coefficient of a drug plays a crucial role in its effective delivery into the brain [5].

It is worth noting that a drug’s particle size significantly impacts its BBB permeability. Small molecules and peptides have been observed to be able to overcome the BBB. Peptides can be either simple or complex, and the folding of their secondary structure largely influences their permeability. This folding hides the charges present on their primary structures, which causes increased lipophilicity [126]. High-flux-dependent drugs will be based only on the cerebral blood flow for their proper delivery into the brain, despite their physicochemical properties; thus, an increased blood flow increases the drug amount that overcomes the BBB [93]. 

There are various methods for delivering drugs to the brain and overcoming the BBB, but most of them are invasive. These methods include the transient disruption of the BBB, infusion through intracerebroventricular or intrathecal routes, the direct injection of the drug into the targeted area, exposure to osmotically active (mannitol) or vasoactive (bradykinin) drugs for BBB disruption, and localized or diffuse exposure to low-intensity ultrasound [127]. These methods pose a significant health risk and could cause major problems such as nerve damage or infection [128,129]. Invasive drug delivery is localized, and the drug concentration in the brain is very low, especially when large molecules are administered [130]. The temporal disruption of brain endothelial TJs by chemical or physical stimuli carries the risk of toxicity and neuronal damage [131]. In experiments where inflammatory cells were used to induce lesions in the BBB, there was a notable decrease in the concentration of occludin and zonula occludens 1 in microvessels, caused by the release of cytokines such as TNF-α, interleukin 1B, and interferon γ [54,132,133]. 

Researchers have conducted studies on improving drug delivery to the brain by enhancing transportation through the endothelial cells. They typically use methods such as increasing the lipophilicity and positive charge levels of a drug, which aid in the passive diffusion and interaction with the anionic glycocalyx. However, these modifications cause a greater nonspecific drug uptake in many tissues, often leading to off-target effects, and in addition, they enhance the drug recognition of efflux pumps [129,134]. A more selective way to stimulate the diffusion of specific small molecules in the brain is to modify them by mimicking the endogenous substrates of the BBB [135].

A more general approach to drug delivery to the central nervous system refers to delivery and transport vectors. Although biological vectors, such as viruses [136,137] and engineered cells [138], have been used to increase transport through the BBB, their safety, permeability across an intact barrier, and brain selectivity are still limited [136,139]. Drug delivery methods usually focus on the vascular BBB, but there may be benefits to targeting the cerebrospinal fluid barrier as well. In both cases, the BBB consists of a single layer of endothelial cells that are linked together by TJs, but has other mechanisms that control or delay plasma leakage through the CNS [140].

## 9. Challenges in Drug Delivery for the Treatment of Brain Tumors

Chemotherapy is a treatment that involves the use of anticancer drugs. These drugs target cancer cells’ DNA, RNA, and protein synthesis or function through different mechanisms. For instance, doxorubicin, an anthracycline, inhibits DNA and RNA synthesis, while bevacizumab antibodies target the vascular endothelial growth factor. Nitrosoureas drugs like carmustine and lomustine interfere with the DNA repair pathways in cancer cells. Chemotherapy can cause side effects like nausea, vomiting, fatigue, and hair loss due to its impact on healthy cells. Other side effects may include changes in taste, a dry mouth, constipation, and a decreased appetite [141,142]. Anticancer drugs have limitations for brain cancer due to the BBB, which prevents most drugs from penetrating it. Only a few drugs are approved by the FDA, such as everolimus, bevacizumab, carmustine, naxitamab-gqgk, and temozolomide [143]. One of the reasons why molecules do not accumulate in the brain is due to the activity of efflux transporters in the brain vasculature, including P-glycoprotein/MDR1 (Pgp), multidrug resistance-associated protein 1 (MRPs), breast cancer-resistant protein (BCRP), and influx transporters like organic anion transporters [144,145,146]. These transporters may efflux substances back into the blood circulation, preventing the accumulation of molecules in the brain [78,147,148]. 

Delivering therapeutic drugs to the brain is challenging due to the BBB. The BBB has TJs made of proteins that seal the gaps between endothelial cells, making it difficult for drugs to pass through. This selectivity restricts the passage of many drugs, as only small lipophilic or gaseous molecules can undergo passive diffusion [76].

Efflux transporters can also remove the drugs that could overcome the BBB, sending them back into the systemic circulation and decreasing the drug concentration in the brain tissues. Metabolic enzymes from the BBB can also metabolize the drugs, reducing their concentration and effectiveness [149]. 

The BBB-overcoming ability of a drug depends on its size, lipophilic properties, hydrogen bonding formation, and molecular weight. Cerebral blood flow also affects drug transport to the CNS [62]. 

The size and electrostatic properties of pharmaceutical compounds determine their ability to cross the BBB. Large, polar molecules struggle to overcome this barrier, while small, lipophilic ones (with a molecular weight lower than 400 Da) have a better chance. However, even smaller molecules have obstacles due to the efflux mechanisms. The spleen removes nanoparticles bigger than 200 nm; liver cells catch those between 100 and 150 nm, and particles under 5.5 nm are eliminated through the kidneys [149]. The BBB can also change under pathological conditions, making drug delivery difficult. Hence, delivering drugs across the BBB remains a significant challenge in treating brain tumors [150].

The charge of nanoparticles affects their ability to cross the BBB. Nanoparticles with a positive charge have an advantage due to the electrostatic interactions with the BBB’s negatively charged proteoglycans [151]. In contrast, neutral particles are less permeable than positively charged nanoparticles by about 100 times [152]. Research studies on rat brains have found that cationic nanoparticles can damage the BBB, while neutral and anionic nanoparticles at low concentrations do not have this effect on barrier integrity [153]. Cationic nanoparticles can cause neuron loss when directly injected via the intracerebroventricular route into the brain [154]. Positively charged nanoparticles can generate reactive oxygen species that damage cells and cause necrosis or apoptosis [155,156]. The BBB endothelial cells’ resistance to anionic charges suggests cationic nanoparticles as a delivery mechanism for negatively charged genetic material, e.g., small interfering RNA, for tumor-targeted gene therapy [157,158,159]. 

The way in which pharmaceutical substances are metabolized and eliminated in the human body affects their concentration in the bloodstream, which can impact their ability to enter the CNS. Certain pharmaceutical substances can bind with blood proteins, which reduces their free concentration and prevents them from overcoming the BBB. The BBB exhibits heterogeneity throughout the CNS, meaning that some regions may have higher or lower permeability than others, resulting in different levels of drug diffusion in the CNS [160]. Researchers are looking for ways to improve drug delivery to the central nervous system (CNS). One approach involves administering high doses of chemotherapeutic drugs through intravenous injections to increase their concentration in the CNS. This method, known as systemic therapy, offers benefits such as consistent drug dispersion throughout the neural axis, regardless of cerebrospinal fluid flow rate or direction. However, it also has drawbacks, such as higher toxicity and the need to exceed a certain drug concentration threshold to overcome the BBB and be effective. Disrupting the BBB can also lead to drug diffusion and penetration into the CNS [88]. 

The infusion of a hyperosmotic solution such as mannitol is the most clinically used approach. This method has been investigated in adult patients with malignant supratentorial gliomas. However, this method is not specific to the tumor, and it is unclear what the exact levels of drug exposure and concentration should be. Other techniques that may be effective include using cytokines or vasoactive substances like bradykinin to disrupt the BBB [161]. However, the use of bradykinin analogs for drug delivery has been abandoned due to its ineffectiveness when combined with carboplatin [162,163]. 

Drugs can enter the CNS through intranasal administration via olfactory or trigeminal pathways via intracellular or extracellular routes. Sensory neurons uptake the drug in the intracellular pathway and send it to the olfactory bulb or shaft. The extracellular pathway can allow molecules to reach the subarachnoid space successfully. Intranasal administration distributes drugs in the olfactory bulb and brainstem [164]. 

Receptor-mediated transport systems enable therapeutic compounds to enter brain tumors by crossing the BBB via receptor-mediated endocytosis and exocytosis. Monoclonal antibody drugs that are targeted at receptors are delivered across the BBB [163]. 

There are specific receptors that can help drugs pass through the BBB. Adenosine receptors have been discovered to aid in drug delivery to the brain by activating A2A receptors or blocking harmful substances and inflammatory immune cells [165]. Glutamate receptors also play a role in the permeability of the BBB, and N-methyl-D-aspartate receptor antagonists have been shown to decrease the permeability. Furthermore, high-intensity magnetic stimulation can increase the barrier permeability and aid in drug delivery by promoting neuronal activity [166]. 

One way to overcome the BBB is to inhibit efflux transporters directly. P-glycoprotein (P-gp) acts as a drug efflux pump, restricting certain drugs from crossing the BBB and entering the CNS. Cyclosporine A is a pharmaceutical agent that can inhibit P-gp’s function. The inhibition of Pgp can affect the clearance of drugs from other organs besides the BBB [167]. 

P-glycoproteins (P-gps) are present in malignant glioma cells and low-grade brain tumors. Modifying P-gp can help to deliver drugs to the tumor area, but first-generation modulators such as verapamil, cyclosporine A, tamoxifen, and some calmodulin antagonists have low binding affinities, requiring high doses and leading to toxicity [168,169]. Second-generation modulators such as dexverapamil, dexniguldipine, valspodar (PSC 833), and biricodar (VX-710) [170,171] have limited success in clinical trials, leading to the development of third-generation modulators, including tariquidar (XR9576), zosuquidar (LY335979), laniquidar (R101933), and elacridar (GF120918) [172,173,174,175]. A research study found that a combination of elacridar with the usual treatment led to a 5-fold increased brain uptake of paclitaxel (PTX) [175]. Other transport inhibitors like sulfinpyrazone, probenecid, and fumitremorgin C have also been reported [163,169,176]. In a study conducted by Tournier and colleagues, it was found that the ABCB1 (Pgp) and ABCG2 (BCRP) efflux transporters in the BBB work together to limit the entry of tyrosine kinase inhibitors. Elacridar is a widely known inhibitor of both ABCB1 and ABCG2, and has been tested in models of central nervous system metastases. In mice, elacridar effectively improved the uptake of erlotinib in the brain. However, similar results were not observed in preclinical human data [177]. In preclinical and clinical studies, elacridar has shown a potential to enhance the brain diffusion of certain drugs such as dasatinib, gefitinib, and sorafenib [178,179,180]. Researchers have also developed a dual inhibitor called Si306, which is twice as effective as dasatinib at inhibiting cancer cell growth and suppressing Pgp activity. Administering the prodrug form of Si306 increased the median survival in mice with GBM tumors [181,182]. A research study conducted by Becker and colleagues explored the use of PI3K/mTOR inhibitors for treating GBM in mice models. The researchers modified two inhibitors, GDC-0980 and its analog GNE-317, to have a lower affinity for efflux transporters. The results showed that both inhibitors had a reduced efflux and a 3-fold higher drug penetration into the tumor core, as well as decreased staining for effector proteins in histology [183]. 

A clinical trial was conducted in pediatric patients with various solid tumors, including brain tumors, and aimed to test the effectiveness of tariquidar (XR9576) in inhibiting Pgp when it was combined with doxorubicin, vinorelbine, or docetaxel. The trial results showed that tariquidar administration increased the tumor accumulation of the Pgp substrate fluorescent dye ^99m^Tc-sestamibi by 22%. Out of the 29 participants, one patient had a complete objective response, and two had partial responses. The toxicities caused by tariquidar were minimal, including transient hypotension, a loss of taste, and nausea. However, when combined with chemotherapeutic agents such as docetaxel and vinorelbine, tariquidar reduced the systemic clearance, leading to increased drug exposure and toxicities. (NCT00011414) [184]. Despite the abundance of preclinical studies investigating Pgp inhibitors, translating them into clinical settings has been challenging. As a result, the search for more potent, selective, and efficacious Pgp inhibitors continues [185].

Various drugs and drug delivery methods have shown potential in crossing the BBB. Nitrosourea drugs, such as carmustine and lomustine, have effectively treated cancerous brain tumors due to their lipid solubility and ability to overcome the BBB. Other commercially available drugs for brain tumor treatment include thiotepa, temozolomide, methotrexate, topotecan, irinotecan, cisplatin, and carboplatin. Additionally, the conjugation of drugs or nanocarriers with ligands that have an affinity for specific receptors presents a receptor-mediated approach to bypass the BBB, allowing for easier entry into brain tumors [159,161]. Peptide–drug conjugates are classified as prodrugs because they link a peptide to a drug using specific linkers. These conjugates usually consist of a cytotoxic agent, a peptide derived from a tumor, and a linker connecting them. In order to develop peptide–drug conjugates, drugs are linked with peptides that can overcome the BBB. The peptide must be able to selectively bind to a specific receptor on the cell surface of the target tissue that is unique or overexpressed in cancer cells and present in sufficient amounts to transport the drug to the tumor. The site and linker of the peptide–drug conjugate must not affect the binding affinity or stability of the target receptor so that it can reach the tumor site and release the drug while minimizing the off-target toxicity. Some commonly used linear and cyclic peptides include arginine-glycine-aspartic acid (RGD peptide) [186,187], gonadotropin-releasing hormone, somatostatin [188], epidermal growth factor, and Angiopep-2 [189]. These peptides are delivered to cells using adsorption-mediated endocytosis/transcytosis, except for Angiopep-2, which enters cells via the low-density lipoprotein receptor-related protein 10 (LRP-1) transporter. These peptides are often associated with cytotoxic agents such as gemcitabine, doxorubicin (Dox), daunorubicin, PTX, and camptothecin [185].

Various methods exist for administering drugs to improve their delivery to brain tumors.

The intracerebral implantation of drugs into the brain has been used in clinical trials, but it is complex and potentially harmful. The implant is made of a biodegradable and biocompatible matrix or reservoir containing the chemotherapeutic agents. It releases drugs over time, but the amount delivered is limited, and reloading may be needed [190]. One example is treating high-grade gliomas with BCNU (carmustine) contained in a polyanhydride polymeric wafer. The drug is released for 2 months, but there is an increased risk of trauma, and other treatments may be more effective [191].

CED, or convection-enhanced delivery, is a method that enables the direct administration of pharmaceuticals to a specific brain region or localized tumor site. This technique utilizes a pressure gradient to increase the drug dispersion in the brain tissue, resulting in precise drug delivery and a controlled spread. Although effective, CED is an invasive technique and requires close monitoring to prevent tissue damage and drug reflux [192]. In neuro-oncology, microdialysis is a well-established technique that has been suggested as a reliable way to deliver drugs directly to tumors. This method allows drugs to diffuse passively across the BBB [193,194], which then distributes the drugs throughout the tumor away from the catheter used for dialysis [163,195].

A non-invasive method to temporarily disrupt the BBB is a focused ultrasound and microbubbles (1–10µm). These microbubbles, contained within gas-filled lipids, are introduced into the bloodstream. It is possible to use these particles as a drug delivery system on their own. For instance, drug molecules can be attached to the shell [196,197,198]. They have also been utilized for delivering stem cells [199] and viral vectors [200]. If microbubbles are provided with a magnetic coating, it can enhance the drug delivery efficiency by keeping them in the target area [201]. The focused ultrasound can then target specific brain areas, causing the microbubbles to undergo oscillations in the acoustic field, generating mechanical forces that exert pressure on the TJs of the endothelial cells, allowing drugs to diffuse more easily into the central nervous system. This effect lasts for a temporary period of 4–6 h [202]. Using microbubbles for drug administration reduces the damage to unaffected brain tissue, but the optimal parameters are still being investigated due to certain risks. There are various approaches for drug delivery, including intra-arterial, intrathecal, intraventricular, intra-tumoral, receptor-mediated transport, the disruption of the BBB, the inhibition of drug efflux, and intranasal administration [163]. Administering drugs directly to a tumor through the bloodstream is known as intra-arterial administration. This method involves injecting medications into the specific arterial vessel supplying blood to the tumor. While it allows for targeted drug delivery, there are some drawbacks to using intra-arterial administration. These include the risk of focal neurotoxicity, the potential for embolism and hemorrhage, and limitations in administering medications to specific areas [161,203]. Studies on administering brain tumor drugs through intra-arterial delivery have shown only slight improvements in patient survival rates [204,205,206,207,208,209]. However, success has been demonstrated by neurosurgeons at New York Presbyterian Hospital/Weill Cornell Medical Center using monoclonal antibodies like bevacizumab. By briefly disrupting the BBB, these antibodies were delivered to the tumor region through intra-arterial delivery [210]. When drugs are administered through intrathecal methods, they have a limited ability to enter the brain’s extracellular space from the CSF [211,212,213,214].

Pharmacokinetics is the study of how drugs affect the body. This includes absorption, distribution, metabolism, and excretion. The method of drug delivery impacts a drug’s bioavailability and duration of effectiveness in the brain. Before absorption, drugs must first be released from their original form, which can vary in duration depending on the drug. Some drugs have a fast-acting effect, while others have a prolonged release, which can impact the onset of their effects and potential side effects. Drug distribution is determined by a drug’s biochemical properties, such as the size, polarity, and binding properties, as well as the patient’s physiology. Regarding the CNS, several drugs are moderately to highly bound to serum plasma proteins. Since only a free drug can act on the target brain tissue, an increased concentration of plasma proteins such as albumin and α-acid glycoprotein can decrease the concentration of pharmacologically active drugs in the body [160]. A drug’s half-life, which represents the time required for 50% of the initial concentration to decay, also influences the final yield and drug concentration in the brain [215].

Active substances with a short half-life are eliminated more quickly than those with a longer half-life. Pharmaceutical drugs can have direct or indirect effects by interacting with receptors, enzymes, or proteins [216]. When drugs are administered, their effects can occur immediately or be delayed depending on the type of mechanisms involved. However, the prolonged use of certain drugs can cause changes in the receptor target, leading to a reduced effectiveness over time. This is called pharmacodynamic tolerance, and has been observed in treating epilepsy and brain cancer. Chronic exposure can cause receptors to be up- or down-regulated, altering their sensitivity and decreasing efficacy. Cancer cells can decrease chemotherapy effectiveness by reducing the target gene expression. Other drug resistance mechanisms include drug pump alterations, detoxification processes, apoptosis, proliferation, and DNA repair [217,218,219].

When a patient has multiple conditions, treating them with medication can be complicated. This is because the ways in which drugs are processed and affect the body can be affected by the presence of other conditions. This can result in drug interactions, which can impact the effectiveness of the treatment. In such cases, it is important to carefully examine the properties of each drug to determine if they can be safely used together [148,220,221,222].

Improving the survival rates for glioblastoma patients requires therapeutic agents to reach active concentrations in non-contrast-enhancing tumor regions. Preclinical and phase I investigators must rigorously evaluate drug delivery to determine new drugs’ therapeutic concentration and diffusion in the CNS. These studies, combined with a better selection of therapeutic agents, can improve the survival rates for patients with hard-to-treat malignancies [223].

## 10. The Albumin Structure and Properties

Albumins consist of a group of non-glycosylated globular proteins, with serum albumins being of the utmost significance. These proteins are found in the blood plasma, are soluble in water, have a moderate solubility in concentrated salt solutions, and withstand temperatures of 60 °C for 10 h [27,224]. Human serum albumin (HSA) has 83 positively charged residues and 98 negatively charged residues. Furthermore, it has a theoretical isoelectric point (pI) of approximately 5.12. The precursor of serum albumin, known as preproalbumin, contains an N-terminus peptide (an extension of amino acids at the N-terminus), which is removed before the protein leaves the endoplasmic reticulum. HSA is a protein produced by hepatocytes in the liver, and the daily amount produced is 9–12 g/day (plasma albumin concentration ranges from 3.5 to 5 g/dL) [225]. Up to 60% of albumin is stored in the interstitial space. Its half-life is 19 days, but it is only maintained for 16–18 h in the bloodstream [226]. Various factors, including hormones such as insulin, thyroxine, and cortisol, influence albumin production. In situations such as hypoalbuminemia, albumin synthesis is stimulated. Conversely, the exposure of hepatocytes to excessive osmotic pressure and high potassium levels can slow down albumin synthesis. The central role of this protein is to maintain the blood’s osmotic pressure and act as the main transporter of hydrophobic molecules (such as fatty acids and hormones), making it a perfect candidate for drug delivery [227].

**Figure 4:**
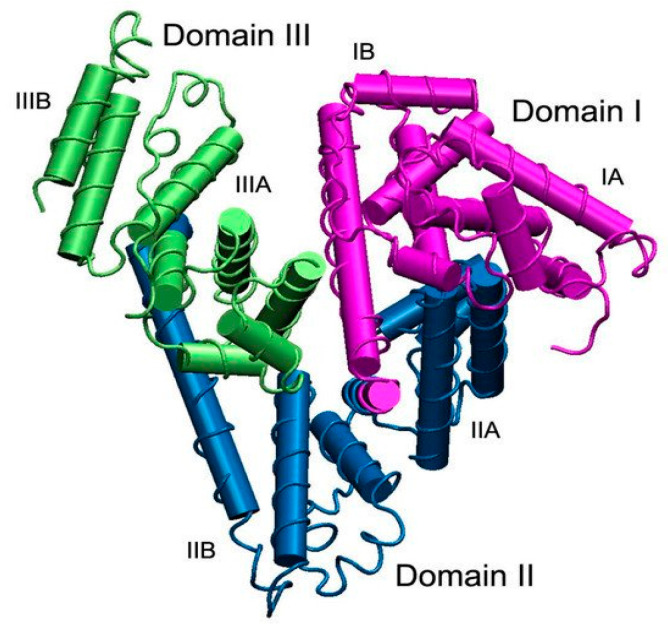
The structure of serum albumin. Domains I, II, and III are shown in purple, blue, and green, respectively; each domain consists of two subdomains, A and B. The albumin molecule does not contain β-sheets and the α-helix and is presented as cylinders [232].

The HSA molecule consists of 585 amino acids, forming a polypeptide chain. The albumin length of the primary sequence may differ in other species, as bovine serum albumin (BSA) has 584 amino acid residues, and rat serum albumin (RSA) has 583 residues. The HSA molecular weight based on the amino acid composition is 66,439 Da; for BSA, it is 66,267 Da, and for RSA, it is 65,871 Da. The secondary structure of albumin contains about 67% helical structures; the remaining 33% are coiled structures and extended chain configurations without any β-sheet configuration [228] (Figure 4). The three-dimensional structure of HSA was discovered quite late, only in the 1990s [229]. A similar BSA structure was obtained in 2012 [230], but the three-dimensional structure of RSA, a product of the main animals used in pharmacological and toxicological experiments, has not yet been obtained. Three homologous domains (I, II, III) [231,232], composed of two subdomains (A, B), form a heart-shaped three-dimensional structure of the protein, which is relatively labile (Figure 4).

Albumin can be functionalized with endogenous and exogenous ligands, water and metal cations, fatty acids, hormones, bilirubin, transferrin, nitric oxide, aspirin, warfarin, ibuprofen, phenylbutazone, etc. [231,232]. The most important human serum albumin binding sites for hydrophobic compounds (especially neutral and negatively charged hydrophobic drugs) are named Sudlow site I and Sudlow site II [233], placed in domains IIA and IIIA, containing hydrophobic domains and positively charged lysine, and arginine residues, respectively [234]. In albumin, site I is also known as the warfarin site because drugs such as azapropazone, phenylbutazone, and warfarin adhere to this domain, and various protein–drug conjugates could be formed. Site II is also known as a benzodiazepine site because compounds such as diazepam, ibuprofen, and tryptophan can interact with this domain. This way, different drugs, such as PTX and docetaxel, can be conjugated and delivered efficiently to the tumor site. Albumin can participate in redox reactions because an albumin molecule contains 17 disulfide bonds and a free thiol group in Cys34 [232].

## 11. Albumin’s Ability to Target Cancer Cells

Blood vessel hyperpermeability and impaired lymphatic drainage, the well-known EPR effect in solid tumors, have been proposed as the mechanisms responsible for the passive targeting of many nanocarriers in solid tumors [235,236]. An essential feature of the EPR effect is the very permeable tumor vasculature, which increases the permeability of the particles with a size of 20–200 nm [237,238]. Because tumors do not have lymphatic vessels, HSA can be extravasated and accumulate in the interstitial space of the tumors [239,240,241]. The defining role of the EPR effect as the mechanism responsible for the passive targeting of delivery nanosystems in solid tumors, even in animal models in preclinical studies, has been questioned [242]. Many studies have focused on drug delivery systems’ accumulation in tumor tissue as a tumor-targeting agent via the EPR effect [243,244]. Albumin can specifically bind to glycoprotein 60 (gp60) and SPARC, thus actively increasing the nanoparticle uptake. This unique absorption mechanism allows albumin-based nanoparticles to overcome drug efflux mechanisms in tumor cells. Studies have demonstrated that nab-PTX (albumin nanoparticles with PTX encapsulated) exhibits a 9.9-time increase in binding capacity to the endothelium, and it was 4.2 times more efficient for PTX delivery compared to Cremophor EL-PTX (a vehicle-based on polyoxyl-ethylated castor oil with PTX incorporated) [27,245,246]. The first nanotechnology-based chemotherapeutic agent and HSA-based product approved by the FDA was Abraxane^®^ (nab-PTX) [247]. In another example, nab-PTX was shown to have improved antitumor activity and tolerability in phase III clinical trials compared to other drugs such as Taxol [248]. In 2005, the FDA approved the use of an albumin-based nanoparticle in nanomedicines for the chemotherapy treatment of breast cancer. In addition, it was authorized for the treatment of non-small-cell lung cancer in 2012 and pancreatic cancer in 2013 [249,250,251,252]. The success of nab-PTX showed the potential of albumin as a drug carrier for imaging and tumor therapies. The mechanism behind albumin accumulation in tumors is not entirely clear. It is uncertain whether albumin infiltrates into the tumors through the EPR effect or if it binds to overexpressed proteins and receptors, leading to its accumulation [245]. Studies have demonstrated that albumin can attach itself to the gp60 receptor with a molecular weight of 60 kDa, also known as albondin, which is present on the surface of vascular endothelial cells and is transported into the tumor interstitium via transcytosis [253,254]. This receptor is found on tumor endothelial cell surfaces, and after the interaction with albumin, it binds to the caveolin-1 protein. Inside cells, the protein triggers the cell membrane to fold inward (invagination of the cell membrane), creating tiny transport sacs called transcytosis vesicles (also called caveolae). These vesicles are responsible for transporting albumin within the tumor [236]. Alternatively, a secreted protein acidic and rich in cysteine (SPARC) can sequester albumin in the tumor stroma and is partially associated with the tumor-specific albumin uptake. SPARC modulates cell–matrix interactions and essential cellular functions such as cell proliferation, survival, and migration [255]. SPARC (also named antiadhesin, osteonectin, BM-40, and 43K protein) is an albumin-binding protein that is overexpressed in various cancer types [256,257]. Research has shown the role of SPARC in albumin uptake in tumors [258,259]; however, this concept is still controversial [260]. SPARC is overexpressed in many types of tumors and absent in normal tissues, interacts with albumin, and contributes to its accumulation within tumors. These two main mechanisms allow the protein to be actively internalized within the tumor cells. There are other albumin receptors, such as gp18 and gp30, in addition to gp60. These are cell-surface glycoproteins with 18 and 30 kDa molecular weights, respectively [261]. They are expressed in the membranes of liver endothelial cells and peritoneal macrophages, and function as scavenger receptors with a strong affinity for damaged albumin [262].

Modified BSA shows a 1000-fold higher interaction tendency for the gp18 and gp30 receptors than native BSA [262]. These two receptors are involved in the endolysosomal sequestration and catabolism of protein, probably as a safety mechanism for the damaged protein (such as those generated via oxidation from inflammation or hyperglycation in diabetes). On the other hand, native albumin does not have a high affinity for the gp18 and gp30 receptors but binds mainly to the previously described gp60 receptor, which is involved in the transcytosis mechanism of albumin through the endothelial cells. Both the gp18 and gp30 receptors interact with modified albumin, which has an altered conformational structure (for example, gold- or formaldehyde-labeled albumin or albumin modified with maleic anhydride) and less with native albumin [263,264]. The cellular uptake of albumin conjugated with colloidal gold particles and maleylated bovine serum albumin has been performed in a manner different from that of native albumin via endocytosis [264]. Modified albumin-based particles have shown a higher affinity for endothelial cells mediated by the gp30 and gp18 receptors, which explains the preferential degradation of modified albumin [265]. 

Previous research has considered that the preferential binding property with modified albumin could be beneficial for the development of drug delivery systems [266]. 

HSA is not immunogenic and is therefore not recognized as a foreign element, but if it is altered or damaged, it is immediately targeted by the immune system and degraded. However, the protein is well known for its prolonged half-life, making it a helpful carrier in drug delivery. The long half-life of albumin is due to protection from intracellular degradation by the FcRn receptor (known as the Brambell receptor), which recycles internalized albumin back into the bloodstream via a pH-dependent mechanism (it has a strong affinity for the low pH of the endosome). 

The FcRn receptor is a transmembrane heterodimeric protein similar to the major histocompatibility complex (MHC) class I, associated with β2-microglobulin (β2-m) [266,267]. The role of FcRn is to bind the proteins (immunoglobulin G-IgG and albumin) in the acidic endosome, preventing them from being degraded in the lysosomal pathway. FcRn and its bound ligand are exocytosed to the extracellular space, where it is released from FcRn at a physiological pH. Albumin is transported from the extravascular space back into circulation through the lymphatic system, making approximately 28 cycles during its lifetime [236,245,268]. 

Studies using confocal microscopy have found that the FcRn receptor expression in the BBB is present in both the choroid plexus and the endothelium [269] and may mediate the efflux of immunoglobulin G (IgG) from the brain into the blood in the process of reverse transcytosis [270,271]. In a murine model of Alzheimer’s disease, it was demonstrated that the FcRn receptor expressed in the BBB was involved in the clearance of immune complexes of IgG, specific for β amyloid peptides in the brains of older mice [272]. The study of the FcRn receptor’s importance in albumin transport across the BBB has not been addressed until now. 

The therapeutic efficacy of small proteins, peptides, and chemical drugs is performed with difficulty due to the short plasma half-life in vivo, with the active principles being rapidly eliminated by the kidneys or liver. Different approaches have been explored to increase the half-life of drugs that have been approved for clinical use and are based on the interactions of active principles with the neonatal Fc receptor (FcRn) or albumin. Albumin’s properties have been explored, and various drug delivery technologies have been used, for example, N- or C-terminal genetic fusion, the chemical coupling of low-molecular-weight drugs, the association of drugs with the hydrophobic domains of albumin, the association with albumin-binding domains that are genetically fused to drugs, and drug encapsulation in albumin nanoparticles [246,273]. Due to the presence of large amounts of albumin at the tumor site and in inflamed tissues, the protein has been first conjugated with drugs and then administered to target the tumor or inflamed tissue [274].

An example is methotrexate conjugated to albumin for the treatment of renal carcinomas and autoimmune diseases such as rheumatoid arthritis [275,276,277]. Another approach is the production of nab-PTX (Abraxane^®^), composed of the lipophilic drug PTX, conjugated to albumin at a high pressure, being used to treat several types of cancer [246]. After administration, the nanoparticles dissociate, and PTX becomes associated with the albumin within the blood. More research is required to demonstrate whether albumin-based nanoparticles could interact with the FcRn receptor. FcRn regulates the HSA half-life in plasma by protecting the protein from lysosomal degradation. Binding is achieved due to the high-affinity interaction of the FcRn receptor with HSA in acidic conditions from the endosomal compartments. Then, HSA is released at a physiological pH after the protein is transported through a recycling pathway to the extracellular surface [246]. Domain DIII of albumin is the main site of interaction with FcRn, and a mutation in this region could result in the formation of a hydrophobic interface and a diminished interaction with the receptor. Thus, the transcytosis of albumin-based delivery systems is reduced, and the intracellular catabolism increases, eliminating albumin fragments and the rapid vascular clearance.

It should be noted that, among the many albumin modifications that are performed at the amino groups in the lysine (Lys) residues (Lys525, Lys195, and Lys233), only Lys525 in domain III decreases the binding affinity of HSA to the FcRn receptor. These results show that the significant structural changes in the modified albumin, especially in the FcRn binding domain, could be used to develop efficient drug delivery systems for cancer treatment [25,269]. One method to specifically bind and stabilize the protein to various drugs is to use the free thiol group from cysteine residue, Cys34, on domain I of albumin through a technology known as the Drug Affinity Complex (DAC^®^) [278]. The pharmacokinetics reflects the power of this technology in humans, where the half-lives of GLP-1 peptide analogs (the peptide similar to glucagon-1) are of several hours, compared to 9–15 days for the same drugs linked to the free thiol group from the cysteine residue (Cys34) in albumin [279]. Another example is an acid-sensitive prodrug of doxorubicin (Aldoxorubicin) that rapidly binds to the SH group of Cys34 after intravenous administration [278]. 

The drug is conjugated through a linker that is cleaved after exposure to an acidic environment, as it is found in the tumor tissues, and aldoxorubicin has been investigated in clinical trials to treat sarcoma and glioblastoma. Whether the acid-sensitive linker is protected or cleaved during FcRn-mediated transport remains to be investigated. A more recent example is the design of ankyrin repeat protein (DARPin) with specificity for the epithelial cell adhesion molecule modified at the N-terminus group by introducing the unnatural amino acid of azidohomoalanine. The modification allows dibenzocyclooctyne to bind specifically to the Cys34 of albumin in a wild mouse. The obtained conjugate could interact with the FcRn receptor in the wild mouse, a strategy that extended the half-life in the plasma of DARPin from 11 minutes to 17.4 h in mice [280]. As discussed above, targeting Cys34 in albumin is unlikely to interfere with FcRn interaction and transport.

Both ligand IgG and albumin bind to the FcRn receptor in a similar pH-dependent manner, which is fundamental to its versatile functions that cover both immunological and non-immunological processes. The FcRn receptor can be found in both hematopoietic and non-hematopoietic cells, including certain types in important organs such as the liver, kidney, and placenta. This highlights the receptor’s importance in regulating the distribution of IgG and albumin ligands throughout the body. It has been discovered that three histidine residues in albumin (His-464, His-510, and His-535) may play an essential role in FcRn binding at acidic pH levels [269,271]. However, additional research is necessary to fully comprehend the functions of FcRn in maintaining brain homeostasis and the potential influence of other albumin receptors [267]. Previous research studies have indicated the potential mechanisms behind the therapeutic benefits resulting from the interaction between HSA and SPARC, a protein that is overexpressed in cancer cells. However, there has been no conclusive evidence to establish a direct link between the accumulation of HSA in tumors and the expression of this protein [281]. Another study showed the binding affinity of BSA to SPARC and the internalization of BSA using a non-cancerous and a cancerous cell line, suggesting that HSA may have an affinity for binding to the SPARC protein that is overexpressed in tumors [282]. Researchers have conducted a study to investigate the accumulation of HSA in U87MG glioma cells through active SPARC-mediated targeting. They also examined the HSA uptake in various cancer cell lines, particularly in SPARC-expressing glioma cells. According to the findings, HSA was uptaken into the tumors, and SPARC contributed to its enhanced accumulation and improved its micro-distribution in gliomas.

Nonetheless, additional clinical research is necessary to validate this theory. An accumulation of HSA in tumors was also observed to decrease 4 h after injection. One possible reason for the decrease in protein accumulation may be a consequence of the tumor cells that use albumin as a source of nutrients (such as amino acids) to support their growth [283].

## 12. Techniques for the Preparation of Albumin-Based Release Nanosystems

Among the many advantages of using albumin as a drug delivery system, we can list the following: a high abundance in blood plasma, immunogenicity, biocompatibility, and biodegradability. Two approaches can be observed for the production of albumin-based drug delivery systems: (1) the chemical binding of drug molecules to albumin molecules to form albumin–drug conjugates and (2) drug encapsulation in nanoparticles based on albumin [284].

Several methods of drug encapsulation in nanoparticles are possible, including self-assembly [285], emulsification [286,287], thermal gelation, nano-spray drying, and desolvation [288]. Among the chemical techniques, desolvation (coacervation), emulsification, and self-assembly are the most commonly used methods. Nano spray drying, thermal gelation, and NAB technology belong to the group of physical obtaining techniques. Reproducibility is a crucial feature to be achieved, and every method used should aim to produce nanoparticles that are characterized by predictable and reproducible properties [236].

**Desolvation** (coacervation) is the most common technique to prepare albumin nanoparticles. Adding a desolvating agent (ethanol or acetone) to an aqueous albumin solution causes albumin dehydration, leading to the formation of nanoparticles. Cross-linking agents like glutaraldehyde (GA) are used to stabilize them, and the reaction occurs over 24 h, leading to the formation of Schiff bases. The average nanoparticle size is 100 nm, but it can vary based on factors like the albumin amount and the cross-linking agent concentration [289,289,290]. Doxorubicin-loaded HSA nanoparticles for tumor treatment have prepared by using the desolvation technique. HSA-based nanoparticles facilitate passive drug-targeting tumors through the EPR effect, overcoming problems associated with drug resistance [291,292]. However, using GA as a cross-linking agent has limitations like toxicity and drug interactions, and the residual aldehyde limits the in vivo applications [284]. Scientists use glutathione to create intermolecular disulfide bonds [284,293] and modify their surface, introducing SH groups using Traut’s reagent. This stabilizes the nanoparticles and functionalized nanoparticles, allowing them to be internalized into cells [294,295]. Figure S1 in the Supplementary Materials schematically shows the synthesis of albumin nanoparticles via the desolvation (coacervation) method, and Figure S2 in the Supplementary Materials shows the process of obtaining nanoparticles based on HSA via a reduction and desolvation method.

Undergoing **thermal gelification (thermally induced aggregation)** causes proteins to unfold and change their conformation due to heat and protein–protein interactions such as hydrogen bond formation, electrostatic and hydrophobic interactions, and disulfide–sulfhydryl exchange reactions [236,296]. The resulting formulation depends on the pH level, the protein concentration, and the ionic strength. This method does not require toxic chemical cross-linking agents [297]. Aggregation is orderly when the pH is higher than the protein’s isoelectric point, and as the pH approaches the isoelectric point, larger aggregates are formed, and spectral deregulation occurs [298]. Albumin-based nanoparticles that co-encapsulated cyclopamine and doxorubicin hydrochloride were prepared using the thermally induced aggregation method. Doxorubicin hydrochloride was mixed with a BSA solution before forming nanoparticles, and then, using the desolvation method, the cyclopamine dissolved in ethanol was added. The nanoparticles were prepared at 65 °C under magnetic stirring (750 rpm) and then ultrafiltered for purification [27,299]. Figure S3 schematically shows the method of obtaining nanoparticles via thermal gelation.

The **emulsification process** is commonly used to prepare polymer micro/nanoparticles. Stirring an albumin solution (aqueous phase) with a non-aqueous solution (oily phase) and a water-soluble surfactant determines the obtaining of a crude emulsion. Homogenization can be achieved with a high-pressure homogenizer. Stabilization can be carried out through thermal gelation or chemical cross-linking [300,301]. BSA nanoparticles were prepared using the emulsion technique with temperature stabilization, and their average diameter was between 400 and 600 nm [302,303]. Also, HSA nanoparticles were prepared using the solvent emulsification–evaporation method, with a size of 130 nm. They were tested in a clinical trial to release the PTX (Abraxane^®^). The solvent can be eliminated through diffusion or low-pressure evaporation, and the albumin nanoparticles can be lyophilized [304,305]. Figure S4 shows the method of obtaining nanoparticles using the emulsification method.

Albumin nanoparticles can be obtained via the **self-assembly technique** using β-mercaptoethanol to increase the hydrophobicity of the protein by cleaving the disulfide bonds or by reducing the amino groups on the surface of the protein through conjugation with a hydrophobic (lipophilic) compound [27,306].

A new self-assembly technique has been developed for encapsulating hydrophobic dye IR780 and docetaxel in HSA-based multifunctional drug-encapsulated nanoparticles. The disulfide bonds in HSA were initially reduced using 2-mercaptoethanol, and then the drug and dye were added to the HSA solution. The nanoparticles obtained were between 140 and 170 nm in size. These new nanoparticles offer significant medical imaging and chemotherapy advantages by combining photothermal and photodynamic therapy with chemotherapy [307]. Figure S5 schematically shows the method of obtaining nanoparticles via self-assembly.

**Spray drying** is a fast and effective method for producing albumin-based micro/nanoparticles in a dry form. It is also a continuous process. The process begins by atomizing the feed liquid and drying the fine droplets formed with hot air or gas in a drying chamber (temperature between 36 and 55 °C). The dry particles are collected using an electrostatic collector. Nano spray drying parameters can be optimized to improve particle properties for specific applications [308]. Figure S6 shows a schematic of the albumin nanoparticle preparation process using the spray-drying technique.

The production of albumin nanoparticles can be effectively achieved through **microfluidic technology**. This method ensures a controlled preparation process resulting in particles with an adjustable size and low polydispersity, making it a reliable alternative for nanoparticle preparation. This technique shows promising potential for large-scale automated pharmaceutical manufacturing. A study conducted in 2020 successfully produced drug-loaded albumin-based core–shell nanoparticles using this technique [301]. The flow system used for the preparation of albumin nanoparticles is illustrated in Figure S7. Intermolecular disulfide bonds that cross-linked the HSA-based nanoparticles loaded with PTX (a biocompatible alternative to GA cross-linking) were produced in a microfluidic platform using glutathione (GSH) to reduce the 17 intramolecular disulfide bonds to free sulfhydryl groups [236,309].

**Albumin-bound (NAB) technology** was introduced as an improved technique for safe and effective systemic drug formulation [310,311]. The FDA approved albumin nanoparticles with encapsulated PTX for cancer chemotherapy. These nanoparticles are obtained through NAB technology via homogenization under high pressure without using surfactants. The nanoparticles that were obtained have a stable structure and a negative charge, and vary in size from 100 to 200 nm. PTX is encapsulated in albumin-based nanoparticles, accumulated in tumors through receptor-mediated transcytosis, and bound to the overexpressed SPARC protein on the tumor surface. According to clinical data, Abraxane (NAB-PTX) presents various advantages over conventional paclitaxel and Cremophor (Taxol). These advantages include lower toxicity, a shorter administration time, and better therapeutic effectiveness [247,310]. It has been reported that Cremophor-EL (Taxol), which was used for systemic PTX administration, caused anaphylactic side reactions in some patients [312]. The preparation of nab-PTX is based on the emulsification and evaporation method [310,313]. The preparation scheme is shown in Figure S8. 

Similarly, gemcitabine-loaded albumin was prepared to treat drug-resistant pancreatic cancer using the same technique. In order to increase its hydrophobicity, gemcitabine was coupled with the myristoyl moiety to produce gemcitabine -C14. The gemcitabine -C14 was dissolved in a chloroform/water solution mixture, and the same technique was used to prepare the nanoparticles as NAB-PTX (Abraxane) [27]. Other drugs such as docetaxel, rapamycin, 17-allylamino geldanamycin, and dimeric thiocolchicine have been encapsulated via the same method, slightly modified [310].

## 13. Delivery Systems Based on Functionalized Nanoparticles Used in Cancer Treatment

Albumin-based delivery systems have been developed for diagnostics and therapy in various medical applications [314,315]. Albumin’s interaction with the FcRn receptor has been studied, and its pH-dependent binding mechanism has been revealed [241,316]. Serum albumin-based delivery systems are considered appropriate for medical use due to several reasons: (1) its molecular weight is above renal clearance, resulting in a prolonged circulation time, which allows for its accumulation in inflamed and malignant tissues; (2) the gp60 receptor, which is found on the surface of endothelial cells, promotes albumin transcytosis and allows for the transportation of proteins to the tumor site; (3) a high albumin abundance and multiple binding sites enhance the pharmacokinetic properties of albumin functionalized with therapeutically active peptides or small antibody fragments. Albumin, being a component of blood, can be utilized as a therapeutic agent in drug delivery applications [317] or a diagnostic marker for various diseases like tuberculosis and acquired immunodeficiency syndrome (AIDS) [318]. Also, albumin can be used as a coating agent for functionalization [319,320].

Researchers are still studying the interactions between drugs and albumins. However, most of the time, the results are inconclusive because, after administration, the plasma proteins can interact with the delivery systems, which can have a negative impact on the drug’s ability to reach its target sites [321]. Its role in drug delivery is determined by the protein corona formation on the surface of the nanoparticles [322,323]. The extensive research on albumin in cancer therapy is due to several factors that lead to its preferential accumulation in tumor cells [324,325]. Cancer is the principal cause of mortality worldwide, and the treatment options include radiation therapy, chemotherapy, and surgery. The protein structure allows for the development of drug delivery systems that could be injected into the bloodstream through intravenous administration. Despite its benefits, albumin-based delivery nanosystems have a few drawbacks. Sometimes, albumin-based nanosystems require, in the preparation stage, the use of toxic cross-linking agents to enhance the stability and avoid swelling and dissolution in vivo, which may otherwise lead to a premature release of the encapsulated drugs before reaching the target tissue [326].

### 13.1. Modification of the Albumin Nanoparticle Surface

**Figure 5:**
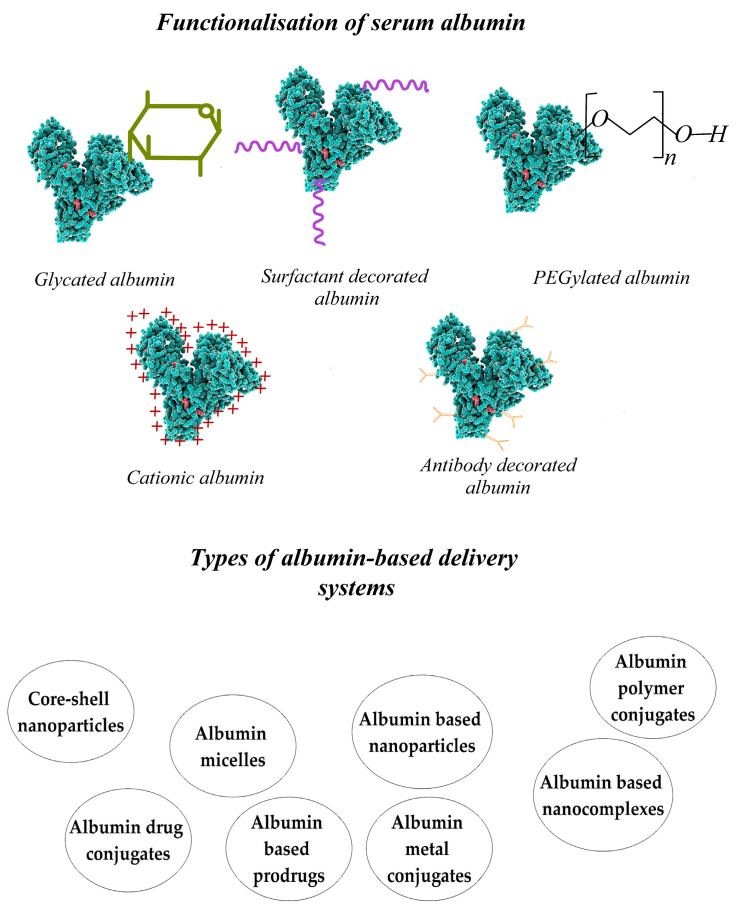
Types of albumin functionalization and the delivery systems based on albumin.

Albumin-based nanoparticles can be easily modified through functional groups, such as the carboxylic, amino, and thiol groups, present on their surface, thanks to the well-defined primary structure of albumin. In order to modify the surface of albumin nanoparticles, ligands are typically conjugated through the formation of covalent bonds with functional groups on the albumin surface. The surface coating and electrostatic adsorption techniques can also be used to modify the nanoparticles’ surface. These modifications enable albumin to serve as a biodegradable carrier for drug release when it is functionalized with ligands. The ligand is used to modify the pharmacokinetic parameters (e.g., surfactants), enhancing the nanosystem stability (e.g., poly-l-lysine), leading to an improved half-life and better bioavailability (e.g., PEG), and slowing drug release (e.g., cationic polymers), or as a targeting agent (e.g., folate, thermosensitive polymers, transferrin, apolipoproteins, and monoclonal antibodies). Table 1 and Figure 5 show some examples of the surface modification of albumin nanoparticles [327].

### 13.2. Albumin Conjugates and Albumin-Coated Magnetic Nanoparticles Used as Theranostic Platforms

Incorporating drugs or genes into HSA or HSA-based nanoparticles significantly improves the therapeutic efficacy of active agents that treat numerous pathological conditions, including neutropenia, hemostasis, and cancer. Albumin-based drug delivery systems can be used for binding with the albondin receptors on the endothelium and SPARC in the tumor interstitium to increase drug accumulation in tumors [246,258,356,357]. Albumin can also aid in transporting molecular imaging agents, allowing for the early detection of diseases and the real-time monitoring of therapeutic responses [273,358,359].

Performing a complete surgical removal of brain tumors is a challenging task, and there is a need for improved techniques to identify the borders of the tumor accurately. These improved methods will help to increase treatment effectiveness while minimizing any collateral damage. One promising option is fluorescence-guided brain surgery, which has recently been accepted as a viable therapeutic option for treating glioma patients. The implementation of this innovative approach can significantly improve patient treatment results by increasing survival rates and reducing disease progression [360,361,362]. Radionuclide-based imaging technologies like single-photon emission computed tomography (SPECT) and positron emission tomography (PET) are commonly used for preoperative imaging and surgical planning [363]. 

A class of imaging agents uses serum albumin as a carrier, specifically albumin-bound gadolinium chelates designed for magnetic resonance imaging (MRI). An example is gadofosveset, or Ablavar [357,364]. Gadolinium chelates, if they are not covalently bound to albumin, can cause the active agent to be released from albumin complexes, reducing the correlation between tumor microvascular parameters determined by the MRI and histopathological data. However, gadolinium chelates that are covalently bound to albumin, such as albumin-(Gd-DTPA), have a longer circulation time, increasing the risk of toxic Gd^+3^ being released into the bloodstream. As a result, albumin-(Gd-DTPA) and its analogs are limited in their use in preclinical studies [365,366]. 

Emulsions and nanosystems containing perfluorocarbon ^19^F (PFCs) are effective MRI contrast agents, but their instability and prolonged retention in organs restrict their use [367,368]. Fluorinated protein conjugates are promising markers for identifying tumor labeling. Mehta et al. [369] produced fluorinated proteins by using fluorinated compounds like S-ethyl trifluorothioacetate or trifluoroacetamidosuccinic anhydride to label amino groups in BSA. These derivatives have proven helpful for NMR imaging and biocompatibility in vivo, although their T2 relaxation times (70 ms) are shorter than those of small, fluorinated molecules (760 ms). The amount of ^19^F labeling that accumulates in a tissue or organ is the most essential factor.

Albumin accumulation in solid tumors is due to the EPR effect, allowing for passive targeting [370]. Two proteins, the gp60 receptor on the tumor endothelium and SPARC in the tumor interstitium, interact with albumin, contributing to its accumulation in tumors [245]. Patients with cancer tend to have lower serum albumin levels because the cancer cells can utilize the amino acids resulting from the digestion of albumin for their nutrition proliferation [371]. Albumin forms a steric barrier in aqueous media that prevents its removal by the reticuloendothelial system in vivo, leading to prolonged blood circulation [327,372]. Albumin conjugates offer several advantages over active agents used in imaging techniques. These advantages include better clearance, intravascular and tissue retention, and the ability to accumulate in tumors. Additionally, albumin conjugates have superior delivery mechanisms. In order to form albumin conjugated with the ^19^F radionuclide, an imaging agent is used. The fluorocarbon molecules must be covalently attached to the protein. The primary amino groups in albumin can provide sufficient functional groups for chemical modification, and its stability at various pH values and temperatures also contributes to easy handling and chemical modification. One effective approach involves introducing fluorine labels into the protein by conjugating them with the free thiol group of albumin cysteine residue or conjugating the fluorinated anhydrides to the ε-amino group of the lysine residue of the protein [369,373].

For a better selectivity of the amino groups in lysine to perform the conjugation with fluorinated anhydrides, the N-homocysteinylation of HSA by the homocysteine thiolactone (HTL) group is recommended as a first step, thus causing the modification demonstrated in vivo to only three of the 59 lysine residues (Lys-525, Lys-137, and Lys-212) [374]. A new conjugate of HTL with perfluorotoluene (PFT) has been synthesized via nucleophilic substitution and characterized. Several fluoro-organic compounds are synthesized via amine arylation. To label albumin with different N-substituted fluorinated HTL derivatives, N-(2,3,5,6-tetrafluoro-4-(trifluoromethyl)phenyl)homocysteine thiolactone (PFT-HTL) was successfully conjugated to the amino groups of HSA. Based on their detection sensitivity, combining the nuclear magnetic resonance (used for preoperative diagnosis and surgical planning) and fluorescence (used intraoperatively) techniques with surgical image guidance is one of the most advanced multimodal molecular imaging techniques. A fluorescent dye (Cy5) maleimide derivative and a fluorinated thiolactone derivative were used to functionalize the albumin, resulting in a dual-label molecular probe for fluorescence microscopy and ^19^F NMR. This synthesized product, PFT-Hcy-HSA-Cy5, was administered to SCID mice with advanced glioma at a dosage of 200 mg/kg, and no toxicity was observed. PFT-Hcy-HSA was found to have a higher intratumor uptake for up to 12 h after injection than in the surrounding healthy brain tissues. According to the ^19^F MRI experiments, there was a higher concentration of PFT-Hcy-HSA-Cy5 in the inner section of the tumor as compared to the outer part. Histology further confirmed this, which also displayed a similar distribution of the fluorescent-labeled albumin solution [357]. PFT-Hcy-HSA-Cy5 accumulation in the outer region of mouse tumors was reduced due to the low vascularization caused by cancer cell necrosis. Some tumor blood vessels have a defective cellular lining composed of unorganized, loosely connected, branching, overlapping, or sprouting endothelial cells [375]. The spaces present among these cells can result in the leakage of tumor vessels, which can provide a pathway for the macromolecular therapeutic agents to reach tumor cells, but may also contribute to disease progression. MRI imaging with PFT-Hcy-HSA-Cy5 can aid in pre-operative diagnosis, surgical planning, and identifying tumor margins through fluorescence. More research is needed to determine if PFT-Hcy-HSA-Cy5 can be used as a probe for diagnosis and imaging, including investigating its toxicity, metabolic fate, and immunological effects [357].

The current research reveals that N-homocysteinylation can cause slight changes in conformation and minor alterations in proteins, resulting in the formation of aggregates that eventually transform into structures such as amyloid plaque over time [376]. 

Overall, when albumin undergoes homocysteinylation, its conformational structure undergoes changes, particularly in the form of a β-sheet conformation. However, a study by Chubarov A. S. et al. in 2015 [357] found that fluorinated albumin maintained most of its α-helix structure. Additionally, it was shown that the N-substitution of HTL can inhibit the aggregation of N-homocysteinylated albumin [357].

A new type of theranostic conjugate was developed, which combines an anticancer fluorinated nucleotide with doubly labeled albumin. The albumin was conjugated using fluorine-labeled thiolactone homocysteine and then linked with the chemotherapeutic drug 5-trifluoromethyl-20-deoxyuridine 50 monophosphate (pTFT). This drug is known to strongly inhibit cell growth [377,378] by inhibiting the thymidylate synthetase enzyme, which is essential for DNA biosynthesis [379]. By inhibiting this enzyme, cancer cells undergo apoptosis. pTFT can also be transformed into its triphosphate form and incorporated into DNA, leading to DNA damage and cell death [380,381,382].

pTFT has been noted for its potential as a chemotherapeutic agent and a promising ^19^F NMR agent. However, when administered as a single agent, its clinical effectiveness is hindered due to its rapid degradation under physiological conditions, non-specific distribution in the body, and quick elimination by the reticuloendothelial system. These factors have led to a plasma half-life of less than 20 min. To enhance the drug’s therapeutic index, researchers have conjugated pTFT with branched polyethyleneimine (PEI) [383]. The cytotoxic effect of PEI has been attributed to the mechanisms resulting from membrane deterioration. Therefore, choosing a different polymer to develop a drug delivery system is very important for successfully designing a polymer system in which the imaging agent is incorporated and used for diagnosis. An optimal delivery system should allow for the efficient release of imaging agents and the release of therapeutic biomolecules, ensuring their optimal distribution to the tumor site [384]. 

A conjugate known as albumin–trifluorothymidine has been utilized to detect and label cancerous tumors. This conjugate contains disulfide and phosphamide bonds that activate the release of the chemotherapeutic agent pTFT when triggered by redox and pH reactions. With this conjugate, cancer can be directly visualized using ^19^F optical and magnetic resonance, and the drug is released in the presence of glutathione. When glutathione is present in tumor cells, it causes the disulfide bond to cleave, releasing the active pTFT drug. It is worth noting that the pTFT’s release from the albumin conjugate is highly sensitive to pH and works best under slightly acidic conditions (pH = 5.4). The PFT-Hcy-HSA-Cy7-pTFT product shows great potential as an optical and ^19^F NMR imaging agent based on in vitro studies. In vitro and primary in vivo studies have shown that these conjugates have the potential for cancer treatment, but further research is needed to fully understand the pharmacokinetics of this HSA-based drug carrier under in vivo conditions [385].

A new theranostic conjugate uses biotin molecules as markers to target specific sites in the body in antitumor drug delivery system applications, which is based on a fluorinated nucleotide anticancer compound, conjugated to biotinylated albumin that is labeled fluorescently for bimodal use. In vitro and in vivo studies have found that the unlabeled PFT-Hcy-HSA-Cy7-pTFT theranostic conjugate had a stronger antitumor effect than the biotin-labeled PFT-Hcy-HSA-PEGBio-Cy7-pTFT conjugate, possibly due to its limited interaction with cellular receptors. Research shows the importance of site-specific modifications for developing albumin-based drugs with desired pharmaceutical properties. In vivo testing has also compared the antitumor effects of two compounds in mice with lung adenocarcinoma and brain tumors. The PFT-Hcy-HSA-Cy7-pTFT compound (without conjugated biotin) had stronger antitumor activity, reducing the tumor volume by 28.2% and 42%, respectively. However, the biotin-labeled conjugate PFT-Hcy-HSA-PEGBio-Cy7-pTFT had a low inhibitory effect on tumor growth. The study found that lung adenocarcinoma tumors decreased by 9.1%, while brain tumors (glioma) treated with the biotinylated conjugate increased by 342%. HSA conjugates that accumulated in tumor tissues were observed through fluorescence-based molecular imaging techniques in combination with computed tomography (CT). The study was conducted on animals in a glioma model, and it demonstrated the accumulation of the fluorescent signal of the compound PFT-Hcy-HSA-Cy7-pTFT in the tumor. 

The compound containing biotin-labeled PFT-Hcy-HSA-PEGBio-Cy7-pTFT did not accumulate in the target tissue. This could be because the reticuloendothelial system quickly removed the modified albumin. By conducting additional research on the structure of albumin conjugates, it could be possible to improve their preparation techniques, leading to new and promising perspectives in the field of drug delivery [386]. Based on mass spectrometry data, it has been observed that the covalent conjugation of HSA with PEGBio molecules resulted in a heterogeneous conjugate mixture, with each having a PEGBio residue attached to a different lysine residue (Lys-536/Lys-560) in domain III of HSA. This may affect the pharmacokinetic properties of the conjugates.

Additionally, the DIII domain of HSA interacts with the FcRn receptor [269,271,387]. Synthesizing albumin conjugates with desired pharmaceutical properties requires the consideration of albumin binding sites. Attaching active molecules to exposed lysine residues can interfere with albumin’s specific receptors on tumor cells, reducing the effectiveness of the therapeutical agent.

The cysteine residue, Cys-34, found in domain I (DI) of the albumin structure, as well as the N-homocysteinylated lysine sites (Lys-525, Lys-205, and Lys-137), can be used to control the conjugation and to develop high-purity drug conjugates with a constant drug charge ratio. Future research should consider the limited studies on how the conjugation to functional groups within albumin can interfere with the binding domain of receptors that are overexpressed in various tumors. By elucidating the interaction between modified albumin and tumor receptors, a more sophisticated design of albumin analogs can be achieved, with a prolonged half-life that maintains its anticancer properties [269,271,387].

Bovine serum albumin and polycaprolactone (BSA-PCL)-based nanoparticles labeled with radioactive iodine can interact with the anti-epidermal growth factor receptor (EGFR) and have been successfully synthesized. In this type of nanoparticle was encapsulated cetuximab, an EGFR inhibitor used for head and neck cancer chemotherapy [388]. The EGFR receptor is located on the surface of tumor cells and is highly important in signaling pathways that regulate cell proliferation, angiogenesis, and tumor metastasis [389,390]. According to a research study, higher levels of EGFR were correlated with resistance to treatment and a poor prognosis for survival [391]. In order to determine cell viability, MTT assays were performed. The results showed that both ^131^I-EGFR-BSA-PCL and ^131^I-BSA-PCL effectively inhibited the proliferation of U251 and U87 glioma cells. However, cells treated with ^131^I-EGFR-BSA-PCL showed a stronger inhibitory effect than those treated with ^131^I-BSA-PCL when exposed to a radiation dose of 0.925 MBq. In vivo, radioiodine imaging studies were conducted on nude mouse xenograft models. The findings revealed that the interaction with the EGFR receptor substantially enhanced the uptake and accumulation of BSA-PCL-based nanoparticles in the in vivo experimental model of nude mice xenografts, and there was an increase in drug release. The potential application of ^131^I-EGFR-BSA-PCL could offer a novel approach to treating glioblastoma [388].

Magnetic nanoparticles have many applications, such as magnetic resonance imaging (MRI), drug delivery, tumor targeting, magnetic hyperthermia, and immune system manipulation. Magnetic particle imaging (MPI) is considered to be a superior imaging technique compared to MRI. Combining the MRI and MPI techniques improves brain signals for the early detection and treatment of brain pathologies [392]. Iron oxide nanoparticles offer great potential for various biomedical applications thanks to their enhanced magnetic properties, large specific surface area, stability, and accessible functionalization possibilities. It is essential to consider the colloidal stability, biocompatibility, and potential toxicity of magnetic nanoparticles in physiological environments when using them in vivo [393,394]. Serum albumin has various applications, including coating drug delivery nanosystems and in the theranostic field. It is biocompatible, circulates in the bloodstream for an extended period, and may help overcome drug resistance in cancer patients. 

Manipulating magnetic nanoparticles with an external magnetic field makes it possible to easily separate them from liquids and direct their targeting within the body. Combining strategies such as local heating, targeted drug release, and MRI monitoring shows excellent potential in chemotherapy and theranostics [395,396,397,398]. Magnetite offers promising properties but is not stable at oxidation and possesses a high surface energy, causing the formation of aggregates. An optimal functionalization of the surface of these nanoparticles can remove these disadvantages.

Protein-coated magnetic nanoparticles are biocompatible, biodegradable, and less toxic [399,400]. Albumin coating reduces unwanted blood component adsorption and improves the targeted active principle release [27,325,326]. Peptides, antibodies, and small molecules have been used as free ligands or attached to nanoparticles to facilitate the delivery to the brain [65,401,402,403]. In receptor-mediated endocytosis, the ligand attaches to receptors present on the surface of vascular endothelial cells, and then the functionalized delivery system is internalized within the cells. The process is called transcytosis, when the ligand/receptor complex is internalized and then released on the opposite side into the parenchyma. As an active targeting reaction, transcytosis can increase the amount of an imaging/therapeutic agent in the parenchyma and thus improve its effectiveness. Transcytosis allows transport across the intact BBB and could be important for treating early-stage disease [392].

Albumin-based delivery systems can be transported across the interior of a cell through transcytosis, which is facilitated by their binding to the gp60, gp30, gp18, and FcRn receptors. This attachment helps to accumulate the delivery system in different tumors, including through the SPARC receptor and its effect [245,358]. A technique used to improve the effectiveness of drugs is the fusion, association, or conjugation of drugs with albumin. When drugs are attached to albumin, it prolongs their circulation time, leading to improved pharmacokinetics and pharmacodynamics of the therapeutics. Albumin has a prolonged half-life because of its large size and interaction with the neonatal Fc receptor (FcRn). This receptor mediates the recycling pathway, which protects the protein from degradation via proteolysis and renal clearance [267,404,405]. 

Magnetic nanoparticles have various uses, including MRI technology, hyperthermia, and drug delivery. However, they have limitations such as a low biostability, toxicity, and tissue specificity, and they tend to agglomerate due to their high surface energy and strong magnetic attraction. High salt concentrations can also affect their colloidal stability, and Fe_3_O_4_ magnetite-based magnetic nanoparticles may lose their magnetism in the presence of oxygen due to oxidation. Surface functionalization can overcome these issues [397,406,407]. When injected intravenously, magnetic nanoparticles form a protein corona by bonding with biological molecules. This results in their fast elimination from the bloodstream [325]. Before intravenous injection, a stable precoating with optimal characteristics is necessary to prevent irregular coating. Organic polymers or low-molecular-weight surfactants are commonly used for coating magnetic nanoparticles [397,406,408,409]. One potential approach is covering these nanosystems with albumin, followed by a subsequent surface functionalization. Serum albumin is an excellent option for obtaining biosensors and butylated nanoparticles that are used in medical imaging and as a theranostic platform [327,410]. The albumin surface can be modified to create intelligent nanosystems with various applications, including medical imaging probes and drug complexes. These nanosystems can interact with albumin receptors to target cancerous tissue. Moreover, the tumor accumulation of these nanosystems can occur passively through the EPR effect, providing additional benefits [25].

Albumin surface modifications include vitamins, vitamin derivatives, carbohydrates, and peptides like RGD and cell-penetrating peptides [411,412,413]. Specific receptors interact with biotin-modified HSA-based nanoparticles, effectively targeting breast and cervical cancer. Conversely, albumin-coated magnetic nanoparticles conjugated with folic acid are used for MRI imaging and specifically target brain tumors [412,414]. Research studies have investigated the use of albumin-coated magnetic nanoparticles conjugated with anti-EGFR and anti-VEGF antibodies [415,416]. These delivery systems have proven to be effective in targeting mammary tumors and brain glioma in mice, demonstrating their potential for in vivo applications [417].

Albumin-coated magnetic nanoparticles are highly stable and ideal for use in animal models. They prevent nucleation and aggregation, even at a high concentration of sodium chloride (0.15 M) [409]. Unlike tannic-acid-coated magnetic nanoparticles, albumin-coated ones maintain their size at different pH ranges and temperatures below 37 °C [311,331] [399,418]. The albumin coating of magnetic nanoparticles enhances their longevity and protects them from non-specific binding with blood components as well as immune system response [25,325,399,409]. Other approaches that use tannic acid, carboxylic acid, and hyaluronic acid can also be used for stabilization and optimal core size formation. Finally, albumin coating is applied to ensure the biostability of magnetic nanoparticles [25].

Before using magnetic nanoparticles in vivo, their toxicity must be studied. The cytotoxic effects of magnetic nanoparticles are caused by several mechanisms, including the release of ferrous ions, the modification of ion channel activity, the dysregulation of gene expression, the disruption of the cytoskeleton, and the formation of reactive oxygen species (ROS) [407]. The MTT test is useful in determining toxicity but does not show non-specific interactions with other blood elements, tissue-specific toxicity, or chronic toxicity. However, a few magnetic nanoparticles have demonstrated acute toxic effects such as inflammation, ulceration, metabolic disorders, and immune response [419,420,421]. The accumulation of magnetic nanoparticles in some organs after degradation can interfere with the physiological iron metabolism, causing damage to mitochondria, cell membranes, and nucleic acids (somatic or inherited mutation) [421]. In order to determine if magnetic nanoparticles can be used in clinical investigations, simple combinations of assays such as plasma stability, ROS formation, and multiple cell lines must be used for ex vivo analysis. Albumin coating usually results in a moderate nanoparticle uptake and low cytotoxicity, determined by ROS production, as many works on in vitro and ex vivo experiments have shown [422,423,424,425]. Their zeta potential must be negative or neutral for optimal protection after coating the magnetic nanoparticles with albumin [426]. Coating drug-loaded magnetic nanoparticles with albumin has been found to improve their therapeutic results in vitro, suggesting a promising possibility for in vivo experiments [25,424,427]. Additionally, albumin coating could help to prevent specific cardiac side effects associated with magnetic nanoparticles [428]. 

Using MRI as a non-invasive diagnostic tool can greatly improve anatomical resolution and detect diseased tissue regions. By utilizing different contrast agents, such as coated magnetic nanoparticles, both the T1 and T2 relaxation processes can be influenced by changes in the water molecule availability near the magnetic core [429]. Albumin-coated magnetic nanoparticles have proven effective in producing multimodal imaging and theranostic platforms. In order to evaluate the potential of this system as a multimodal imaging technology, the surface of the albumin-coated magnetic nanoparticles was labeled with a ^64^Cu-DOTA complex (for positron emission tomography, PET) and a fluorescent dye (Cy5.5). Triple imaging techniques (PET, near-infrared fluorescence, and MRI) were successfully tested on a glioma mouse model [430].

One important goal is to develop theranostic platforms by using magnetic nanoparticles coated with albumin. These platforms possess considerable potential in the treatment of drug-resistant cancer. Researchers have designed a delivery system for PTX based on magnetic nanoparticles coated with albumin, which can potentially be used for diagnosis through MRI techniques and in drug-controlled and targeted release applications [431]. Albumin-coated magnetic nanoparticles loaded with doxorubicin, methotrexate, curcumin, or curcumin/5-fluorouracil have shown promising outcomes in various cell lines and animal models [25]. An excellent therapeutic effect was also obtained on rat models with gliosarcoma tumors [25,432]. The integration of hyperthermia and chemotherapy has been demonstrated ex vivo in different cell lines using PTX [423] and etoposide (topoisomerase-II inhibitor) [433]. Composite albumin nanoparticles containing magnetic nanoparticles with encapsulated etoposide were developed. These nanoparticles are intended for use in ex vivo testing, specifically to evaluate the effectiveness of the combined hyperthermia and chemotherapy methods.

Magnetic hyperthermia uses magnetic nanoparticles to destroy cancer cells with heat generated by converting energy from an externally applied magnetic field. Normal cells can withstand the heat, while cancer cells cannot survive above 43 °C. The method shows promise for destroying cancer cells in areas with magnetic nanoparticle accumulation [434]. Scientists have developed a delivery system based on magnetic nanoparticles covered with HSA to deliver etoposide, a DNA-damaging cancer drug that promotes cancer cell death. The nanoparticles were prepared using a modified co-precipitation method. Testing on U87 glioma cells showed a significant decrease in cell viability when exposed to alternating magnetic fields and heat. Using an etoposide-loaded HSA-based delivery system significantly reduced the viability of U87-MG cells to 7.8% when combined with heat treatment. According to the findings, the viability of the cells decreased to 59.4% with only heat treatment and 53.8% for the combined treatment with free etoposide. This delivery system can aid in treating brain tumors and MRI imaging. Magnetic hyperthermia is also a promising treatment option [433,435].

Carmustine (BCNU) is commonly used in the treatment of brain cancer. However, its short half-life and lack of selectivity often result in the need for systemic administration, leading to severe adverse reactions such as hepatotoxicity, bone marrow suppression, and pulmonary fibrosis [436,437,438,439]. Scientists have developed a nanoprobe based on albumin-coated superparamagnetic iron oxide (SPIO), carmustine (BCNU), and indocyanine green (ICG) that can diagnose and treat glioblastoma multiforme (GBM). It can be used for bimodal imaging and controlled drug release, and the obtained nanoparticles were functionalized on the surface with Angopep-2 polypeptide (TFFYGGSRGKRNNFKTEEY) to target the low-density lipoprotein receptor-related protein (LRP) present in BBB and GBM cells. The delivery nanosystem obtained was encoded with ANG-BSA/BCNU/ICG magnetic nanoparticles. These nanoparticles are stable, spherical, and have magnetic properties, with an average diameter of 85 nm. The obtained delivery nanosystems are biocompatible, have mild side effects, and selectively target tumors while having controllable drug release.

The encapsulated magnetic nanoparticles also exhibit good biocompatibility, colloidal stability, and the ability to overcome the BBB and selectively target GBM cells [440]. The literature mentions that lactoferrin-modified polymeric nanoparticles containing BCNU and tamoxifen can be directed to the brain to treat GBM and have antiproliferative effects on GBM [441,442,443]. ICG has also been evaluated for its ability to target GBM and to overcome the BBB using in vitro and in vivo NMR/fluorescence bimodal imaging techniques and tested in the ANG-BSA/BCNU/ICG magnetic nanoparticle delivery system to inhibit tumors. During in vitro and in vivo experiments, it was observed that the ANG-BSA/BCNU/ICG magnetic nanoparticle could inhibit tumors more efficiently compared to the control group through in vivo experiments [444,445,446]. After performing a cytotoxicity analysis, it was found that the treatment with the magnetic nanoparticles-ANG-BSA/BCNU/ICG had a higher inhibitory effect on the GBM cell line (U87MG) than the control samples. This innovative delivery system with co-encapsulated BCNU and imaging agents provides an effective strategy for targeted therapy and the intraoperative localization of GBM. The ANG/BSA/BCNU/ICG magnetic nanoparticles inhibit the proliferation of GBM cells more than BSA/BCNU/ICG magnetic nanoparticles or free BCNU and can be used for both diagnosis and treatment, representing a highly valuable theranostic nanoplatform [440].

Nanoparticles provide a promising solution for drug-resistant cancer by serving as a theranostic platform for developing new drugs for bimodal applications such as therapy and diagnosis. It represents a novel approach to developing next-generation medication by manipulating its physical, chemical, and biological properties. This technique includes the targeted release of active ingredients, controlling the size, and surface functionalization. Magnetic nanoparticles show potential as a core for theranostic platforms. The obtained nanoparticles can be used in MRI diagnostic imaging, manipulated with an external magnetic field, and have a hyperthermia effect. Coating magnetic nanoparticles with albumin enhances their stability, allows for a targeted release in tumors, and improves their biocompatibility and biodegradability. This results in a significant accumulation in cancerous tissue due to the EPR effect and receptor binding ability [25]. 

## 14. Albumin-Based Delivery Systems That Overcome the BBB and Treat Glioblastoma

GBM is a very aggressive form of brain cancer that accounts for 47% of all brain cancer cases. It has high invasiveness, poor clinical prognosis, frequent recurrence, and high mortality rates. Various delivery systems, including nanoparticles, have been developed to deliver chemotherapeutic drugs such as docetaxel [447,448,449], PTX [450,451,452], doxorubicin [453], or other small molecule chemotherapeutics [454,455,456,457]. These delivery systems also have encapsulated antibodies [458,459], RNA [460,461,462], or peptides [463] in the hope of improving GBM therapy. Despite extensive research, there has not been enough progress in developing a delivery system to treat glioblastoma effectively. Nanocarriers used for this purpose are typically made of synthetic materials, which tend to accumulate in the liver and spleen, causing significant side effects. Additionally, these nanocarriers cannot overcome the BBB. However, protein and viral nanoparticle-based delivery nanosystems have shown promising results in targeting and transporting bioactive compounds across the BBB, providing hope for future treatment options [464]. 

Scientists have created GBM-targeting synthetic protein nanoparticles by combining polymerized HSA and oligo(ethylene glycol) (OEG) functionalized with iRGD31, a cell-penetrating peptide [465]. The siRNA-loaded nanoparticles have the ability to be internalized within the cells and block the function of STAT3 (a signal transducer and activator of transcription 3). HSA was chosen for the obtaining of nanoparticles due to its quick elimination mechanisms, proven clinical relevance, and compatibility with therapeutic agents and original peptides. Albumin-based delivery systems have also been shown to interact with cell surface receptors such as SPARC and gp60, which are overexpressed on glioma cells and tumor endothelia [465,466,467,468].

**Figure 6:**
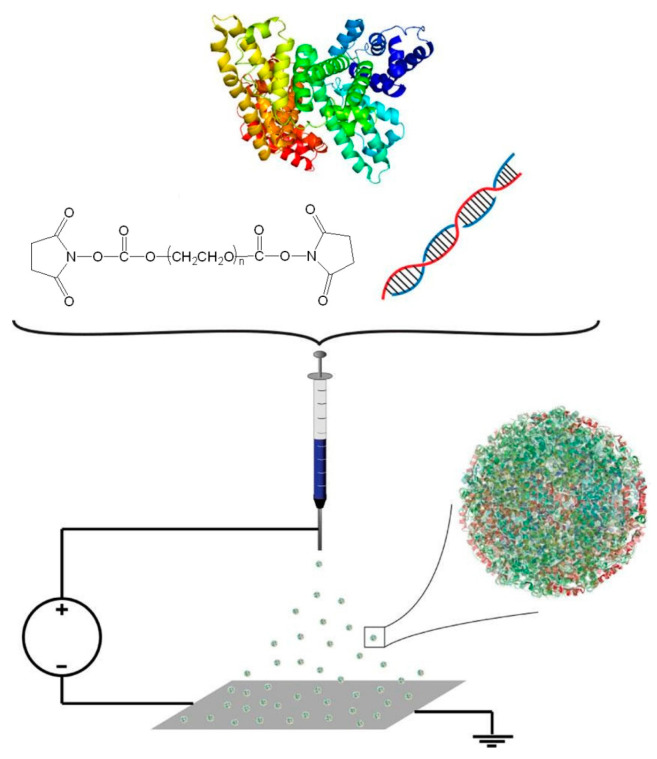
The schematization of the process used to obtain nanoparticles through the electrohydrodynamic jet method [470].

The preparation process of albumin nanoparticles involves electrohydrodynamic jetting, which uses the atomization of dilute polymer solutions to produce well-defined nanoparticles [469]. By using this method, the size of the delivery systems is considerably reduced, allowing for the quick evaporation of solvents and the solidification of non-volatile nanoparticle components. The procedure involves dissolving HSA in a mixture of ethylene glycol and ultrapure water, where bifunctional-OEG (NHS-OEG-NHS) is added to the HSA solution. The iRGD peptide is then incorporated into the nanoparticles and added directly to the jet solution. For siRNA nanoparticles, siRNA was complexed with branched polyethyleneimine, and the mixture was added to the jet solution. The control nanoparticles did not have siRNA encapsulated. The solutions were pumped through a syringe with a 26 G needle at a flow rate of 0.1 mL/h for the final jet, while a constant voltage (7.5–9.0 kV) was applied. The particles were placed in aluminum dishes and then incubated for seven days at 37 °C to ensure complete polymerization. After purification and harvesting, the particles were kept at 4 °C in the dark. Figure 6 schematically shows the obtaining process of these nanoparticles. Their average size was 115 ± 23 nm in the dry state and approximately 220 nm in the swollen state [470]. 

Nanoparticles with iRGD peptide helped to treat aggressive intracranial GBM tumors in mice. The nanoparticles were then distributed throughout the tumor mass, and the nanoparticles with siRNA encapsulated were administered to inhibit STAT3 without surgery. The nanoparticles were combined with focused radiation therapy for longer-term survival in mice, even with a second induced tumor (87.5% of the mice showed improvement in the treatment against GBM, achieving longer-term survival). Based on these findings, the delivery system used is a successful nanosystem for the specific delivery of encapsulated biological substances. When used together with the current standard-of-care methods, the nanoparticles that deliver the biological compound for STAT3 inhibition provide a favorable immunomodulatory response, particularly in the highly aggressive and recurrent GBM disease model. Additionally, there were only minimal indications of liver toxicity and no notable variations in blood cellular components, which are linked to the function of the liver and kidneys, implying that no noticeable off-target side effects occurred as a result of the treatment [470]. 

The iRGD peptide interacts with integrins, binds to neuropilin-1 (NRP-1), and activates an endocytotic/exocytotic transport pathway [471]. In order to enhance iRGD-mediated tumor localization, it can be administered co-encapsulated in nanoparticles [472] or attached to the surface of nanoparticles through covalent binding [473]. In vitro tests have shown a reduction in STAT3 protein expression with free siRNA, but not in an animal model. Nanoparticles have proven to be highly effective in treating tumors by combining the benefits of proteins and synthetic nanoparticles. They can efficiently deliver therapeutic agents into tumors through systemic administration.

Moreover, they have the potential to eliminate resistant cancer cells long-term by utilizing immunomodulatory proteins immobilized in delivery nanosystems. In a highly aggressive intracranial tumor model, 87.5% of mice survived long-term [470]. 

The nutrient transporters on the BBB are important for maintaining normal brain functions by transporting essential nutrients such as amino acids/peptides, sugars, and proteins. These transporters can also be used as entryways for drug delivery into the brain. Researchers have explored various transporters such as LAT-1, GLUT-1, LDL, and transferrin receptors for this purpose in recent decades [474]. 

Albumin is an essential source of nutrients for the body. However, albumin is typically prevented from entering the brain. When tumors grow rapidly and require more nutrients, they use albumin as a source of amino acids and energy. This significantly increases the albumin supply in tumor tissues [283,475]. The process of internalizing albumin in tumors is facilitated by albumin-binding proteins like SPARC and glycoprotein 60 (gp60). These proteins are responsible for endothelial transcytosis and endocytosis in tumor cells [245].

A study proposed the use of albumin-binding proteins that were overexpressed in glioma as drug carriers. To achieve this, researchers developed a delivery system using functionalized albumin nanoparticles with increased permeability in the cell matrix. These nanoparticles contained co-encapsulated PTX and fenretinide (4 HPR). In order to functionalize the nanoparticles, low-molecular-weight protamine (LMWP) was used to form covalent bonds with the sulfhydryl group in albumin. The LMWP-modified albumin-based nanoparticle system was designed to co-administrate PTX and 4 HPR, both used in brain cancer therapy. One benefit of co-encapsulating PTX and 4-HPR is that their hydrophobic and synergistic properties trigger the self-assembly of albumin into nanoparticles. Although PTX and 4-HPR are poorly soluble in aqueous solutions, they can be successfully encapsulated in albumin-based delivery systems.

This study presents a new delivery system for brain tumor treatment that improves drug release through active targeting and the co-encapsulation of two drugs with synergic effects within BSA-based nanoparticles functionalized with LMWP (a peptide that increases cellular permeability). The functionalized nanoparticles overcame the BBB, were internalized into the cells, and infiltrated into the brain tumor, where the drugs were released. 

**Figure 7:**
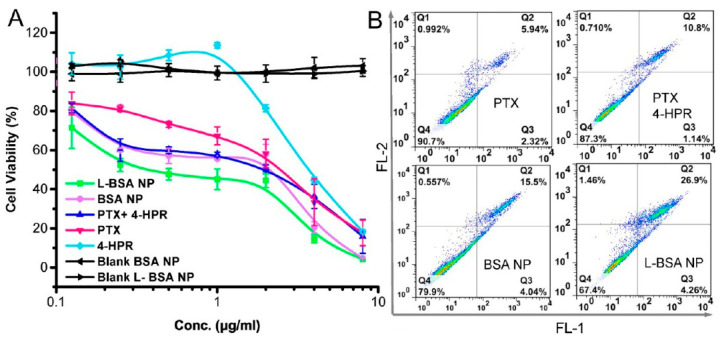
Effect of albumin nanoparticles on U87 cells. (**A**) MTT test for nanoparticles with U87 cells for 48 h. (**B**) Cell apoptosis assay at a combined dose (2 μg/mL for each drug) [479].

A green self-assembly method for producing nanoparticles using denatured BSA (in the presence of urea/NaBH4) with two encapsulated drugs, PTX and 4-HRP, has been developed. This method involves using a highly concentrated urea solution and reducing conditions (NaBH4) to diminish non-covalent interactions like hydrogen bonds, as well as the hydrophobic effect. The protein unfolds and forms a linear structure when the albumin disulfide bonds are cleaved. Lipophilic drugs interact with the protein’s hydrophobic domains, which induces BSA’s self-assembly into nanoparticles. The formation of disulfide bridges further stabilizes the nanoparticles. This process eliminates the need for toxic cross-linking agents and energy consumption. LMWP is derived from protamine sulfate and is obtained via enzymatic digestion with thermolysin. Drug release strategies from LMWP-functionalized delivery systems have been used to overcome various bio-barriers such as the skin, intestinal mucosa [476], intra-tumoral heterogeneity, and transporter-mediated drug efflux [477]. LMWP-functionalized drug-albumin conjugates overcame the drug-resistant efflux and exhibited an enhanced anticancer treatment efficacy [478]. The size of the obtained albumin nanoparticles was less than 150 nm. Figure 7 shows the effect of albumin nanoparticles on U87 cells [479].

LMWP enhanced the intra-tumoral infiltration of drug-loaded nanoparticles, which was also in accordance with other studies [477]. The study was conducted to assess the efficacy of LMWP-functionalized and non-functionalized BSA nanoparticles in inhibiting the growth of U87 cells, and it was found that the effectiveness of the treatment was dependent on the dosage used (as shown in Figure 7A). Both types of nanoparticles with co-encapsulated drugs exhibited better cytotoxicity than the mixture of free drugs. The LMWP-functionalized BSA-based nanoparticles demonstrated superior antitumor activity compared to the non-functionalized BSA-based nanoparticles. The percentage of apoptotic cells was 15.1% for the non-functionalized BSA nanoparticles and 24.6% for the LMWP-functionalized BSA nanoparticles, compared to 10.8% for the free drugs (Figure 7B). The tumor epithelium and glioma cells overexpressed albumin-binding proteins such as SPARC and gp60. These overexpressed proteins are primarily responsible for delivering nanoparticles in brain tumors that mimic biomolecules. LMWP enhanced the cell permeability, and the LMWP-functionalized BSA-based nanoparticles overcame the BBB. The functionalized nanoparticles could infiltrate within the tumor, and the cellular uptake was improved. The albumin nanoparticles modified with LMWP and containing two drugs, PTX/4-HPR, have proven to be effective in stopping tumor growth in subcutaneous and orthotopic glioma models. Their effectiveness lies in their ability to target various mechanisms, including anti-angiogenesis, apoptosis, and the regulation of the tumoral immune microenvironment [479].

While cytotoxic medications like nitrosoureas and platinum-based drugs may decrease the proliferation of glioma cells, they can also result in severe side effects, such as nephrotoxicity with the use of cisplatin and pulmonary toxicity using nitrosoureas [480,481], which causes poor tolerance and limited efficacy [482]. Effective treatments for glioma are essential because the tumor cells proliferate rapidly and use a lot of energy to maintain abnormal growth. An abnormal energy metabolism is a critical feature of glioma [483,484,485,486]. Adenosine triphosphate (ATP) is the cells’ primary energy source. Interestingly, glioma cells are more sensitive to ATP levels than normal cells [487]. Research has shown that restricting energy, specifically ATP, can effectively inhibit glioma cells’ growth [488,489], suggesting that inhibiting ATP formation in tumor cells may be a helpful strategy for glioma therapy.

Glioma cells generate ATP mainly through a glycolytic pathway, known as the Warburg effect, rather than through oxidative phosphorylation. Inhibiting glycolytic enzyme activities can significantly reduce ATP production and tumor cell proliferation [490]. However, when glycolysis is inhibited, tumor cells increase ATP synthesis through the mitochondrial pathway to maintain normal functions [491]. Therefore, monotherapy with glycolysis inhibitors alone may not be sufficient for effective treatment [492].

A drug called Albendazole (Abz) can inhibit the functions of glycolytic enzymes, suppress the expression of hypoxia-inducible factor I, and ultimately inhibit glycolysis [493,494]. Additionally, silver nanoparticles can reduce mitochondrial function and inhibit ATP generation through the mitochondrial pathway [495,496,497]. Therefore, the most effective way to inhibit ATP synthesis would be to block both the glycolytic and mitochondrial pathways simultaneously. 

In the practice of traditional Chinese medicine, aromatic substances like borneol, musk, and corn mint are known as messenger drugs. They are used to direct other drugs to specific organs, particularly the brain. These substances act as targeting ligands for brain drug delivery systems. They can also increase permeability through the BBB by reducing the expression of TJ proteins, which is essential for specific drug delivery [498,499,500,501,502]. Since menthol does not have any reactive functional group to conjugate with BSA, its analog, namely para-mentha-8-thiol-3-one, having the skeleton structure of menthol, was used for protein functionalization. For albumin functionalization, 2-iminothiolane was allowed to react with the amino groups in BSA to introduce sulfhydryl groups. Then, BSA thiolate and para-mentha-8-thiol-3-one were cross-linked with 1,4-butanediol diglycidyl ether to form thioether linkages. The functionalization degree of albumin with menthol was 62.30%. Functionalized and non-functionalized proteins were first denatured with Tris (2-carboxyethyl) phosphine (TCEP), and then ATP inhibitors, Abz, and silver nanoparticles were added to this solution to be encapsulated. Nanoparticles were obtained via precipitation by adding ethanol–ethyl acetate solution (1:1, *v*/*v*). TEM images showed that a protein corona covered the encapsulated silver nanoparticles [503].

After modification with menthol, the zeta potential of the nanoparticles became more negative than those that were not functionalized, while their amine groups were reduced. Nanoparticles containing silver nanospheres have a significantly higher drug loading degree (DL%) compared to those without silver nanoparticles. This is due to the larger specific surface area of the silver nanospheres [504].

Albumin nanoparticles modified with para-mentha-8-thiol-3-one successfully delivered drugs to glioma tumors in the brain by overcoming the BBB. The tests conducted both in vitro and in vivo showed that co-encapsulating albendazole and silver nanoparticles in functionalized BSA-based nanoparticles inhibited ATP synthase by targeting both the glycolytic and mitochondrial pathways. This led to cytotoxic effects such as inhibiting cell proliferation, causing cell cycle arrest, and inducing apoptosis in tumor cells. Menthol-modified albumin nanoparticles were also effective in inducing apoptosis in tumor cells and were found to be safe for normal cells without causing organ toxicity in vivo. These findings suggest that this delivery system could be used in clinical trials [503].

A significant issue with using an albumin-based drug delivery system is its weak structural stability due to its natural characteristics and the complex in vivo environment containing numerous proteins and enzymes [505]. However, under an oxidative atmosphere, the intermolecular bonds can be regenerated, leading to the reassembly of the albumin molecules into relatively stable nanoparticles and an improved ability to encapsulate drugs in the hydrophobic domain [506]. Research has shown that tumor cells have a much higher level of glutathione (GSH)—which can cleave the disulfide bonds—at 10 mmol/L, compared to normal cells with only 0.2 mmol/L. These results suggest that the intermolecular disulfide bonds that are present in albumin molecules could effectively self-crosslink and stabilize drugs, while allowing for redox-sensitive drug release in tumor cells [293,507]. In order to effectively treat glioblastoma, nanoparticles need to be functionalized to overcome the BBB and the BBTB. The selective overexpression of neurokinin-1 (NK-1) receptors has been observed in several malignant tumors, including glioma [508]. The SP peptide, which has the sequence Arg-Pro-Lys-Pro-Gln-Gln-Phe-Phe-Gly-Leu-Met, is a ligand that binds to NK-1 and can be effectively used as a targeting ligand in HSA-based nanoparticles. HSA molecules were functionalized with the SP peptide using a standard reaction between a maleimide and thiol groups from albumin. PTX-loaded HSA nanoparticles were prepared using a desolvation method, which was stabilized by the intramolecular disulfide bonds. In order to prevent rapid clearance by the immune system in vivo, it was determined that excessive functionalization with the targeting peptide fragment could cause issues. Thus, the optimization of the functionalization degree with the SP peptide was achieved at approximately 50% [507].

The obtained SP-HSA-PTX nanoparticles, with a spherical structure and an average diameter of 150 nm, have a 7% drug loading degree and a 90% encapsulation efficiency. A minimal release of PTX molecules from the nanoparticles in PBS was observed due to the intermolecular disulfide bonds. Exposure to glutathione (10 mmol/L) significantly increased the release rate. The cross-linked SP-HSA-PTX nanoparticles, remained stable in the extracellular environment, preventing drug loss and reducing toxicity in normal tissues. The SP-HSA nanoparticles were found to have a better cellular uptake on BCEC and U87 cells than HSA nanoparticles, suggesting that both cells overexpressed the NK-1 receptors, which recognize the SP peptide. As a result, the SP peptide has a dual-targeting ability to bind to glioma cells and overcome the BBB [507].

**Figure 8:**
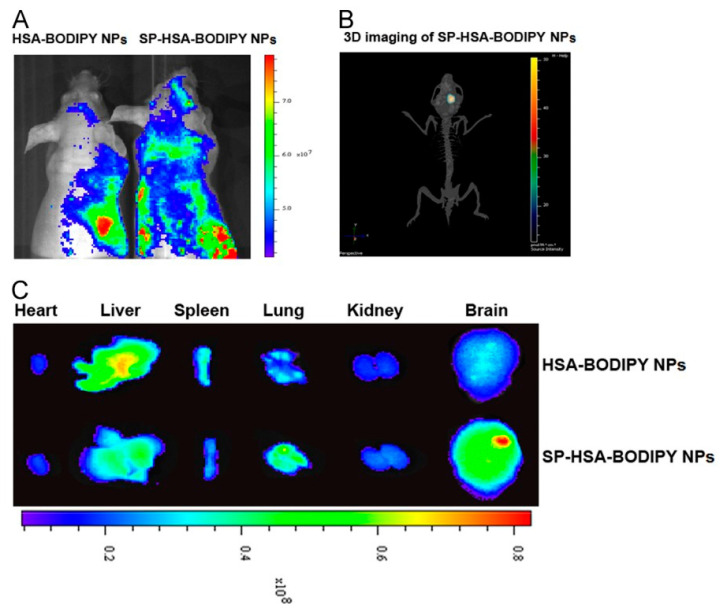
After intravenous administration, in vivo and ex vivo distribution of HSA-BODIPY nanoparticles and SP-HSA-BODIPY NPs. Images were taken 24 h after injection (**A**), and 3D images were taken 24 h after intravenous injection of SP-HSA-BODIPY nanoparticles (**B**). Representative ex vivo images of brains and organs from mice sacrificed at 24 h **(C**) [509].

In order to evaluate the targeting impact of nanoparticles on glioma, several albumin nanoparticles loaded with BODIPY were injected intravenously into mice that had developed U87-Luci cancer cell-induced tumors. The nanoparticles were then monitored using an in vivo imaging system. Figure 8 demonstrates that mice receiving SP-HSA-BODIPY functionalized nanoparticles had a more noticeable fluorescent signal in the tumor region within 24 h of after injection than those receiving non-functionalized HSA-BODIPY nanoparticles. Figure 8C further indicates that the group treated with SP peptide-functionalized nanoparticles displayed a weaker fluorescent signal in the liver than those who received non-functionalized albumin nanoparticles, indicating a reduced liver toxicity. The distribution of nanoparticles in the heart, spleen, lungs, and kidneys was comparable in both groups [509].

Tests conducted in vivo showed that the SP-HSA-PTX nanoparticles were the most effective in treating tumors, compared to the HSA-PTX nanoparticles and Taxol nanoparticles. This effect is likely due to the nanoparticles’ ability to accumulate PTX at the tumor site while reducing the systemic adverse effects. The mice group treated with the HSA-PTX nanoparticles also had a longer survival time. It is likely that the nanoparticles’ increased accumulation through the EPR effect, combined with albumin’s initial targeting ability, contributed to the observed outcome. Additionally, albumin serves as a source of nutrients and energy for the rapid growth of tumors [245,283]. Apart from delivering effective therapy, safety is an essential aspect of an ideal drug delivery system. After observing the histological status of the heart, liver, spleen, lung, and kidneys, it was determined that the group treated with the SP-HSA-PTX nanoparticles did not experience any significant toxic or pathological changes [509].

A new controlled drug release system was developed by researchers using cationic BSA (CBSA) that was attached (conjugated) to the surface of poly(ethylene glycol)-poly(lactide) nanoparticles (PEG-PLA) [510]. This system is ideal for delivering drugs to the brain. In order to obtain the conjugated nanoparticles, the CBSA was thiolated, and then it was covalently conjugated to the functional groups of polyethylene glycol (PEG) located on the PEG-PLA nanoparticles via a maleimide reaction. The size of the nanoparticles produced was between 80 and 83.5 nm on average. 

In order to evaluate the transcytosis and any potential toxicity induced on the BBB, assays were conducted using brain capillary endothelial cells (BCECs) and astrocytes in a co-culture model. The permeability of the BBB was measured using ^14^C-labeled sucrose. The results indicate that nanoparticles caused high paracellular resistance. The BBB permeability using the CBSA-based conjugated nanoparticles in vitro was calculated and compared to that when using BSA-based conjugated nanoparticles. The TEER in the co-culture model was measured at 313 ± 23 Ω.cm^2^. At a concentration of 200 μg/mL, the permeability of the ^14^C-labeled sucrose remained the same as that of the CBSA nanoparticles, indicating that the integrity of the tight endothelial junctions within the BBB was not affected by the CBSA nanoparticles used. Studies have demonstrated that excessive amounts of free CBSA can inhibit transcytosis, and nanoparticles made from CBSA have exhibited lower toxicity toward BCECs. The cationic albumin CBSA-based nanoparticles had a permeability rate of approximately 7.8 times higher than that of the BSA-based nanoparticles [510].

Table 2 presents a list of albumin-based delivery systems that can be used to treat brain tumors by overcoming the BBB. This table also highlights the key features of each delivery system.

## 15. Clinical Trials

Nanocarriers show promise in treating brain tumors, but no new nano-drug has been approved yet for brain tumor therapy. New nanomedicines have a low approval rate (less than 10%) due to safety and efficacy issues in preclinical and clinical trials. Regulatory agencies require manufacturers to conduct thorough preauthorization studies to assess new nanomedicines’ quality, safety, and efficacy. However, finding suitable preclinical models that accurately represent human conditions is a major challenge that impedes the clinical translation of nanomedicine [44].

There is ongoing and consistent research on the advancement of albumin-based nanoparticles to treat brain cancer. Researchers are exploring alternative administration methods aside from intravenous, and discovering new applications for diagnostic purposes. Currently, clinicaltrials.gov only lists two clinical trials related to using albumin nanoparticles in treating glioblastoma. Albumin-bound Abraxane, or PTX, is the first albumin-based delivery system to have undergone clinical trials. Abraxane utilized albumin’s natural affinity for hydrophobic drugs to encapsulate PTX at multiple sites within its structure [304]. Albumin–drug interactions are hydrophobic and do not involve covalent bond formation. However, there may be some cross-linking degree between albumin molecules on the nanoparticle surface [304]. The nanocomplexes are typically 130 nm in diameter, and hydrophobic interactions are generated through a synthetic process involving high-pressure homogenization. This process involves mixing drug and albumin molecules in an aqueous solution and then subjecting the mixture to high pressure as it passes through narrow spaces in a homogenizer [526]. 

Abraxane is a medication that not only provides a safer formulation for PTX but also offers significant benefits in terms of pharmacokinetic properties, rapid drug distribution, and an increased volume of distribution. This is achieved by taking advantage of the EPR effect. It is worth noting that the degradation of the complex in the bloodstream happens quickly and can result in single albumin molecules bound to PTX. Endothelial receptors like gp60 can regulate albumin transport, promoting the caveolae-mediated translocation of albumin from the blood vessels’ lumen to the subendothelial space. The FDA and EMA have approved Abraxane for various types of cancer, including metastatic breast cancer [527], locally advanced or metastatic non-small-cell lung carcinoma (NSCLC) [304], and the first-line treatment of metastatic pancreatic adenocarcinoma [252]. Clinical investigations on pancreatic cancer focused on the cysteine-rich secreted protein acid SPARC, which confirmed a correlation between Abraxane and SPARC expression [528,529,530,531]. Clinical trials (phases 3 and 4) have demonstrated that Abraxane is more effective when combined with other drugs, such as atezolizumab, GEM (gemcitabine), and carboplatin against triple-negative breast cancer; with GEM against pancreatic cancer [532] and melanoma [533]; and with carboplatin against NSCLC [534]. However, when combined with bevacizumab, serious side effects were recorded [535,536,537,538,539,540]. Abraxane also enhances the effects of biological therapy, such as the TLR-7 immune modulator activator imiquimod [541,542]. Additionally, Abraxane was tested against non-Hodgkin’s lymphoma [543], with the delivery system covalently coated with rituximab to induce toxicity to CD20-positive cancer cells [544].

Nab-Rapamycin, also known as Abi009, uses the technology from Abraxane to inhibit mTOR, affecting cancer cell viability. Encapsulation in albumin complexes has proven to be effective in delivering highly hydrophobic molecules in this case, and the same approach can be used to deliver other drugs with similar physical and chemical properties and mechanisms of action. The studies that have been conducted so far have only been tested against advanced carcinomas with mTOR mutations. The aim was to evaluate non-toxic doses of AB009 to treat bladder cancer in phase 1/2 trials. All conditions that were tested displayed a good tolerability [545]. There are ongoing trials to test the effectiveness of ABI009 alone or in combination with other drugs against sarcoma [546] and various pediatric solid tumors, such as central nervous system cancer [547]. Researchers have also tested albumin nanoparticles loaded with sirolimus, an mTOR inhibitor, against glioblastoma [548].

A new device with ultrasound emitters was implanted in patients with recurrent glioblastoma during the surgical resection of the recurrent tumor. The device temporarily and reversibly opened the BBB before chemotherapy perfusion with albumin-bound PTX. The concentration of the drug was measured in different parts of the removed tumor. Carboplatin was administered together with albumin-bound PTX to certain patients who participated in this phase I clinical trial. This study aimed to determine a safe and efficient dosage for albumin-bound PTX, examine the effect of BBB opening on PTX concentration in tumors, and estimate the effectiveness of this treatment in reducing tumor size and extending life. The BBB can be opened using low-intensity pulsed ultrasound and intravenous microbubbles (LIPU-MB). MRI was utilized to investigate the opening of the BBB before and after sonication. The study was conducted for a total of 11.89 months. Some patients experienced side effects such as encephalopathy and grade 2 peripheral neuropathy due to dose-related toxicity at 260 mg/m^2^. BBB opening caused by LIPU-MB was often linked to mild to moderate headaches that were temporary in nature, affecting 71% of patients. The most frequent adverse events during ultrasound treatment were neutropenia, leukopenia, and hypertension, affecting 47%, 29%, and 29% of patients, respectively. After conducting pharmacokinetic analyses, it was found that LIPU-MB increased albumin-bound PTX or carboplatin concentrations in the brain tissue. This was achieved using the implantable ultrasound device in the skull that temporarily opened the BBB, allowing for the safe and repeated delivery of cytotoxic drugs (such as PTX or carboplatin) into the brain. As a result, the average concentrations of these drugs in the brain tissue increased. The success of this study led to a phase 2 trial combining LIPU-MB with albumin-bound PTX and carboplatin, which is currently ongoing [549].

## 16. Conclusions and Perspectives

The most prevalent type of brain cancer is GBM, which is known to be aggressive and invasive. To treat GBM, patients typically undergo surgical resection, radiation therapy, and chemotherapy. However, the main challenge in administering drugs for treating GBM is the BBB. This review detailed the primary mechanisms that enable drugs to cross the BBB: transporter-mediated transcytosis and receptor-mediated endocytosis. The BBTB is formed only when clusters of tumor cells grow to a specific volume, and the BBB deteriorates. This literature review focused on understanding the factors that influence the passage of drug molecules through the BBB. It was established that the overexpression of various proteins plays a crucial role in drug diffusion. A drug’s physicochemical properties, such as lipophilicity, hydrogen bond formation, size, and surface charge, as well as the protein binding capacity, cerebral blood flow, clearance, and barrier integrity, are essential. Several research papers have shown that serum albumins can bind specifically to 60 kDa glycoprotein (gp60), leading to its uptake into cancer cells through transcytosis.

Additionally, serum albumins can bind to SPARC (an acidic and cysteine-rich protein) and prevent the efflux mechanisms of the drug, leading to a better absorption of nanoparticles in the tumor. Also, the significance of the neonatal Fc receptor in prolonging the half-life of drugs encapsulated in albumin-based delivery systems was highlighted, and the specific domains within albumin where the neonatal Fc receptor interacts were discussed. The main site of interaction in albumin with FcRn is domain DIII. If this region undergoes a mutation, it could lead to the formation of a hydrophobic interface and a diminished interaction with the receptor. This literature review briefly discusses some techniques for preparing albumin nanoparticles. A major drawback of cross-linking albumin nanoparticles is the utilization of GA. GA can lead to severe hematological side effects and bind to a drug’s functional groups, which cannot be released at the therapeutic dose in the tumor site, determining inadequate chemotherapy. Albumin nanoparticles have amine, carboxylic, and thiol functional groups. They can be modified with various ligands to achieve specific properties like improved stability and prolonged systemic circulation. These nanoparticles can also target specific receptors for transport through the BBB, or accumulate in the tumor. Using albumin nanoparticles is a promising method for treating human glioblastoma. These nanoparticles are up to 200 nm in size and can encapsulate drugs, genes, growth factors, or inhibitors for the receptors present in various tumor signaling pathways. To improve the stability of nanoparticles and the efficacy of the drugs involved, further in vitro and in vivo studies are needed. Albumin is a promising candidate for the conjugation of radiopharmaceuticals and for coating magnetic nanoparticles that are used in the theranostic field. It can provide biocompatibility, prolonged blood circulation time, immunogenicity, and low toxicity. A supplementary analysis of the cytotoxicity of magnetic particles is required because most of the research has been conducted on cell lines using the MTT assay. In order to ensure the stability of the plasma and avoid the formation of reactive oxygen species, it is essential to assess the impact of magnetic nanoparticles. It is also recommended to use multiple cell lines for the MTT assay. Developing theranostic platforms using albumin-coated magnetic nanoparticles or albumin-conjugated radiopharmaceuticals is a challenging goal. These platforms should contain various therapeutic agents that can be used for both magnetic resonance and fluorescence imaging for diagnosing and treating drug-resistant brain tumors. 

In order to improve the targeting of brain tumors, drug delivery systems should be optimized with ligands that are specific to the receptors that are overexpressed in these tumors. This approach will prevent non-specific toxicity and ensure the safety of nanomedicine. While the FDA has approved albumin nanoparticles with PTX (Abraxane) for treating other types of cancer, this treatment option has not yet received approval for brain cancer. Research has demonstrated that albumin-based nanoparticles have the potential to overcome the BBB and effectively target brain tumors. However, clinical trials are necessary to translate these findings into improved chemotherapy treatments for glioblastoma multiforme (GBM), ultimately enhancing patient survival.

## Figures and Tables

**Table 1:** Surface modification of albumin nanoparticles.

Classes of Substances Used for Modification	The Functionalized Serum Albumin-Based Nanoparticles’ Characteristics	Active Principle	Mechanism	Refs.
Surfactants Polysorbate 80	Encapsulating the drug in these delivery systems reduces its toxicity and increases its AUC while decreasing the distribution volume, clearance, and cardiotoxicity.	Doxorubicin	Cover	[328,329]
Cationic Polymers Poly(ethylene imine)-PEI	These functionalized albumin-based nanoparticles with PEI have numerous benefits, including protection against enzymatic degradation, the lack of a need for toxic cross-linking agents, the modification of the surface charge, reduced plasma protein adsorption, and facilitated in vivo applications. However, PEI may exhibit slight toxicity in cells.	Bone morphogenetic protein-2 (BMP-2)	Cover	[330,331]
PEI/Poly(ethylene glycol) or PEG	BSA-based nanoparticles, functionalized with PEI and PEG, show reduced toxicity in cells of PEI and improved biocompatibility. Coated BSA nanoparticles promote bone structure formation and exhibit improved physicochemical properties.	Bone morphogenetic protein-2 (BMP-2)	Cover	[332]
Poly-L-lysine (PLL)	Functionalized albumin nanoparticles with PLL have improved stability in water, which is directly proportional to the molecular weight and the concentration of PLL.	Bone morphogenetic protein-2 (BMP-2) and siRNA	Cover	[333,334]
Thermosensitive polymers Poly(N-isopropyl acrylamide-block-polyallylamine)(PNIPAM-AAm-b-PAA)	BSA-based nanospheres functionalized with PNIPAM-AAm-b-PAA release adriamycin less efficiently than unconjugated nanospheres at 37 °C. However, drug release efficiency increases at higher temperatures, such as the cloud temperature, due to the solubilization of the polymer, suggesting that the nanospheres can be targeted to tumors with slightly higher temperatures than the body’s physiological temperature.	Adriamycin	Conjugation of PNIPAM-Aam-b-PAA to the carboxyl groups of albumin nanospheres using the carbodiimide (EDC) reaction.	[335,336]
PEG PEG/mPEG succimidyl propionate	PEGylated albumin nanoparticles have numerous benefits, including prolonged systemic circulation, and increase the half-life of 5FU by 50 times. They also reduce immunogenicity and promote the nanoparticles’ accumulation in tumors through the EPR effect. Amino groups in albumin nanoparticles were PEGylated using mPEG succinimidyl propionate.	5-Fluorouracil (5-FU)	Pegylation of BSA was performed by succinimidyl propionate-activated mPEG through their free amino groups.	[337]
	Reduces immunogenicity.			
Polyethylene glycol)-poly (thioether amido acid)-polyethylene glycol); methoxy poly(ethylene glycol)	HSA-mPEG nanoparticles had a lower drug loading efficiency than HSA due to limited binding sites. The surface modification resulted in a slower drug release in the presence of enzymes due to a hydrophilic steric barrier on the nanoparticle’s surface.	Rose Bengal (RB)	Grafting	[338]
FOLATE Folic Acid	Folate receptors are often overexpressed in human cancer cells and can be used to target the nanoparticles at the tumor sites. Folic acid, used to functionalize albumin-based nanoparticles, is stable, inexpensive, and non-immunogenic compared to other options. By binding to cell surface folate receptors, it can be internalized through receptor-mediated endocytosis, making it an effective marker for directing drugs to cancer cells. Folate-conjugated albumin nanoparticles represent a drug delivery system that shows specificity for cancer cells.	Doxorubicin, PTX, cisplatin, vinblastine sulfate, mitoxantrone, and epigallocatechin-3-gallate.	The carboxylic group of folic acid was covalently conjugated to the amino groups on the surface of albumin nanoparticles using the 1-ethyl-3-(3-dimethyl aminopropyl) carbodiimide (EDC) coupling technique.	[339,340,341,342,343,344,345,346]
			adsorption on the surface of albumin nanoparticles.	
Peptides arginine-glycine-aspartic acid (RGD)	The cyclic peptide RGD is a ligand with a high binding affinity to integrin αvβ3. The inhibition of integrin protein avb3 from binding to their specific ligands causes apoptosis in endothelial cells in newly formed blood vessels. Peptides that mimic the ligands of these integrins and anti-integrin antibodies can inhibit their ligand binding.	5-fluorouracil	Conjugation	[347]
Arginine–Alanine–aspartic acid (RAD)	BSA-based nanospheres that are sterically stabilized and functionalized with RGD and RAD peptides have been developed to target tumor vasculature specifically.	5-Fluorouracil		
	The functionalized BSA-based delivery systems with these two peptides have proven to be much more effective in preventing lung metastasis, angiogenesis, and tumor regression than free 5FU, unfunctionalized BSA particles, or RAD-functionalized ones.			
RGD	The drug delivery system was developed based on PEGylated HSA-based nanomicelles obtained via self-assembling and coated with cyclic RGD peptides. The delivery system was tested by incubating it with human melanoma cells (M21+) that expressed αvβ3 integrin and showed an increased drug uptake and retention in these cells.	Doxorubicin	Conjugation	[306]
	The HSA-based delivery system also facilitated the rapid release of the drug through specific mechanisms in endosomes and lysosomes.			
CREKA (cysteine–arginine–glutamic acid–lysine alanine)	Researchers have discovered a peptide called CREKA, which can attach to clotted plasma proteins present in tumors. By utilizing this peptide to functionalize Abraxane nanoparticles, the accumulation of PTX in tumors can be enhanced, leading to better therapeutic outcomes. When antitumor treatment was carried out with CREKA-functionalized micelles, it was observed that there was no significant difference in comparison to the treatment with untargeted Abraxane.	PTX	Coupling of the peptides to Abraxane via their cysteine sulfhydryl group using a sulfo-SMCC (sulfosuccinimidyl-4-[N-maleimidomethyl] cyclohexane-1-carboxylate) crosslinker.	[348]
LyP-1i (Cys-Gly-Gln-Lys-Arg Thr-Arg-Gly-Cys)	The accumulation of nanoparticles occurred in the tumor’s blood vessels, resulting in the formation of aggregates that encompassed red blood cells and fibrin.			
	Abraxane is the albumin-based nanoparticle with an average diameter of 130 nm, in which the drug PTX has been encapsulated.			
	Abraxane nanoparticles functionalized with LyP-1 peptide could be transported to extravascular sites, resulting in a significant growth inhibition of tumors when compared to untargeted and CREKA-conjugated Abraxane.			
	This technique allows for the precise targeting of nanoparticles to tumor tissue, resulting in superior treatment.			
Apolipoproteins Apolipoprotein E (Apo E, A-I, B-100),	HSA nanoparticles with functionalized Apo E (covalently linked) can be uptaken into the brain endothelial cells through endocytosis after intravenous (i.v.) injection into the bloodstream.	Loperamide	Covalent linkage formed and used as a bifunctional Mal-PEGNHS cross-linker that reacts with an amino group on the surface of HSA particles as well as a thiol group introduced into Apo E	[349,350]
	Some of these functionalized particles can penetrate the brain parenchyma, but this can only be achieved through transcytosis to overcome the BBB. TJs in brain endothelial cells are not opened or modulated, and the nanoparticles have not been observed in association with TJ complexes or in the paracellular space.			
	Polysorbate-coated nanoparticles seem to deliver drugs to the CNS in a similar way as they uptake in blood circulation, as the nanoparticles functionalized with Apo E or AI after intravenous injection.			
	Apolipoproteins that modified the nanoparticles in mice had significant antinociceptive effects within 15 min of injection, lasting over an hour, unlike the loperamide solution.			
Transferrin Transferrin -SPDP	The PEGylated albumin nanoparticles were functionalized with transferrin via a coupling reaction with maleimide-poly(ethylene glycol)-N-hydroxy succinimide. These nanoparticles were prepared using a nano-emulsification technique and glutaraldehyde cross-linking.	Azidothymidine, FITC-dextran	The BSA particles were coupled with thiolated transferrin at the distal end of the PEG chain.	[351]
	The obtained nanoparticles overcome the BBB through amino acid transporters and can be used as drug delivery systems, although the drug immobilization efficiency decreases for transferrin-functionalized nanoparticles. The functionalized nanoparticle’s size is slightly larger than non-modified ones and varies from 114 nm to 124 nm.			
Transferrin receptor monoclonal antibodies (TfR-mAb)	In order to prepare the functionalized HSA nanoparticles with transferrin, a heterobifunctional cross-linker NHS-PEG-MAL-5000 was used for SH group activation, followed by adding a thiolated transferrin solution to react with the activated sulfhydryl group. The nanoparticles’ size ranged from 155 to 188 nm.	Loperamide	TfR-mAb was covalently linked to HSA nanoparticles for functionalization.	[352]
	When HSA-based nanoparticles containing loperamide were functionalized with transferrin or TfR-mAb, the drug’s ability to cross the BBB was noticeably improved, allowing it to enter the brain. These functionalized nanoparticles loaded with loperamide demonstrated powerful antinociceptive effects. However, the nanoparticles functionalized with immunoglobulin G2a (IgG2a) could not transport this drug across the BBB.			
Monoclonal antibodies specific humanized anti-HER2 antibody, trastuzumab (Herceptin^®^)	The trastuzumab-conjugated HSA nanoparticles were utilized to target HER2-overexpressing cells in patients with metastatic breast cancer. The experiments demonstrated effective internalization via endocytosis, dependent on time and dosage.	Antisense oligonucleotides (ASOs) against Plk1 (Polo-like kinase 1).	The covalent binding of the monoclonal antibody took place at the sulfhydryl groups of HSA using a bifunctional compound poly(ethylene glycol)-α-maleimide-4-NHS for their activation.	[353,354]
	The trastuzumab-conjugated HSA nanoparticles were found to attach to the surface of HER2-overexpressing cells, including BT474, MCF7, and SK BR-3. Following incubation with trastuzumab-modified HSA nanoparticles, the delivery systems significantly reduced both Plk1 mRNA and Plk1 protein expression.			
cetuximab, DI17E6	A monoclonal antibody known as DI17E6 has shown promise in preventing the proliferation of cancer cells with the epidermal growth factor receptor (EGFR) overexpressed on their surface. It inhibits angiogenesis and can be used for cancer therapy. Encapsulating doxorubicin in DI17E6-functionalized albumin nanoparticles improves cytotoxicity.	Doxorubicin	Covalent binding to HSA nanoparticles.	[355]

**Table 2:** Functionalized albumin-based nanoparticles that overcome the BBB.

Type of Drug Delivery System	Obtaining Methods	Diameter (nm)	Functionalization	Mechanism of Action on the Tumor and Overcoming the BBB	Specific Features of the Drug Delivery System
Nanoparticles based on BSA (BSA Nps) were cross-linked using glutaraldehyde (GA), and then the temozolomide (TMZ) was encapsulated [511,512].	Desolvation	167–261	Using EDC and NHS, carbodiimide chemistry was employed to conjugate hyaluronic acid (HA) or chondroitin sulfate to BSA-based nanoparticles.	Targeting through the CD44 receptor.	In vitro tests show that BSA-based nanoparticles can overcome the BBB and inhibit U87 MG cell growth. Moreover, these nanoparticles also stimulate the production of reactive oxygen species inside the tumor cells.
					Their uptake is facilitated through endocytosis, specifically the caveolae pathway. The CD44 receptor is responsible for directing the nanoparticles to the tumor site.
					In vivo studies show improved pharmacokinetics and brain accumulation of TMZ-loaded nanoparticles compared to the free drug.
					The biodistribution studies on TMZ-loaded BSA-based nanoparticles revealed a greater concentration of TMZ in the brain, while its levels in important organs like the liver and lungs were notably reduced.
Albumin nanoparticles having encapsulated LY2157299 that inhibit the TGF-β I receptor (TGFβRI) and celastrol, an mTOR pathway inhibitor [513].	Emulsion	126.8	DCDX (cgreirtgraerwsekf) mixed with albumin.	Nicotinic acetylcholine receptors.	Biomimetic nanoparticles can repolarize tumor-associated macrophages (TAMs) from the M2 to M1 phenotype by inhibiting the STAT6 pathway, decreasing TGF β1 secretion, and causing cell apoptosis.
					It was found that the treatment effectively blocked the TGF-β/SMAD2 signaling pathway. Moreover, the use of nanoparticles significantly increased the survival rate and reduced the proportion of M2-type TAMs, TGF-β1, and lactic acid levels in glioma tissues.
Nanoparticles based on HSA that are cross-linked with GA and contain encapsulated acidic temozolomide (TMZA) [514].	Desolvation	111.7–177.5	-	Uptake/accumulation in cells.	The optimized nanoparticles contain 4 mg of TMZA with 0.05% sodium cholate, resulting in a 111.7 nm size and 5.5% loading degree. The nanoparticles did not cause a decrease in cell viability, and the drug release from them was quite rapid. The optimized nanoparticles demonstrated a remarkable cellular uptake after being incubated with glioblastoma cell line GL261 and BL6 brain cancer stem cells for 24 h.
BSA-based nanoparticles cross-linked with GA containing co-encapsulated two drugs, PTX, and chloroquine diphosphate salt (CQ) (autophagy inhibitor) [515].	Desolvation	51–53	Folic acid conjugation on nanoparticle surfaces can be achieved through a reaction with carbodiimides DCC (N,N′-dicyclohexylcarbodiimide) and NHS (hydroxysuccinimide).	Mechanism of autophagy inhibition.	In vitro, the combination of PTX and CQ therapy resulted in a higher occurrence of cell apoptosis than treatment with PTX alone.
					Encapsulated PTX caused the overexpression of cancer stem genes (SOX2, POU5F1, and NANOG) in glioma cells. But using nanoparticles containing chloroquine decreased their expression. Autophagy is significant in this process.
					Out of all the delivery systems, the one containing two co-encapsulated drugs was the most effective in inducing cell apoptosis.
Drug delivery systems based on cationic HSA and HSA modified with mannose having doxorubicin encapsulated [516].	High-pressure homogenization technique.	90.5 ± 3.1	In order to obtain cationic HSA, ethylenediamine was linked to HSA through an EDC reaction. Additionally, HSA was modified with mannopyranoside using a thiol-maleimide reaction.	The nanoparticle uptake mechanism uses dual cationic absorptive transcytosis through the glucose transporter pathway.	The doubly modified nanoparticles exhibited the highest efficiency level in terms of transportation through the bEnd3 mouse endothelial cell monolayer and in U87MG glioblastoma cells.
					The c/m-HSA nanoparticles showed a higher level of localization in cerebral glioma than the native HSA-based nanoparticles.
					The enhanced effectiveness in treating glioma appeared to result from a system combining dual cationic absorptive transcytosis and glucose transportation using both c- and m-HSA.
Album lipid nanoparticles with encapsulated docetaxel [517].	Desolvation	110.1± 40.2		Enhanced permeation and retention effect (EPR).	The lethal dose of albumin lipid nanoparticles containing docetaxel was found to be 180.6 mg/kg, which is 75.3% higher than that of Taxotere^®^.
					The obtained nanoparticles have been proven to be effective in preventing the proliferation of several cell lines, such as U87, A549, Raw 264.7, and bEnd.3, and can even induce cell apoptosis.
					In addition, when used in vivo, imaging has shown that docetaxel, which is encapsulated in albumin lipid nanoparticles, can be located and accumulated at the glioma site. This delivery system can inhibit tumor growth and prolong the median survival time in mice.
Doxorubicin was encapsulated within PLGA nanoparticles that were coated with dendrimers that contain cationized albumin [518].	Reaction with carbodiimide (EDC).	156 ± 10.85	EDC reacts with BSA’s carboxyl group to generate a reactive O-acylisourea intermediate that rapidly reacts with the dendrimer’s amino group to create an amide bond.	The anticancer mechanism through caspase-mediated apoptosis.	The release of doxorubicin encapsulated in nanoparticles depends on the pH, reducing the hemolytic toxicity and increasing the drug uptake into the cells. The ex vivo test results show that the nanoparticles lead to cytotoxicity in U87MG glioblastoma cells and an increase in the expression of the caspase-3 gene (about 5.35 times), resulting in an anticancer effect.
HSA-based nanoparticles in which PTX was encapsulated [519].	NAB technology	140		The mechanism that inhibits gene expression in glioblastoma cancer cells.	Researchers conducted a study to investigate the impact of combining miR-34a with albumin-bound PTX nanoparticles on anti-tumorigenesis in glioblastoma cell line U251. The results indicated a significant decrease in cell viability when miR-34a was combined with albumin-bound PTX nanoparticles.
					Furthermore, the treatment of U251 cells with miR-34a and PTX-containing nanoparticles led to a considerable inhibition in SURVIVIN gene expression compared to those treated with miR-34a alone or drug-free nanoparticles.
Encapsulation of aclarubicin in cationic BSA-conjugated PEG nanoparticles surface [520].	Emulsion (o/w)	50–58	Maleimide is used to react with thiolated PEG nanoparticles to graft cationic BSA onto their surface.	The treatment mechanism involves opening the TJs in the BBB and accumulating the nanoparticles at the tumor site.	Cationic BSA-conjugated PEG nanoparticles labeled with 6-coumarin (fluorescent probe) accumulate more in tumor mass than unconjugated ones 24 h post-intravenous injection.
					Functionalized nanoparticles released a higher drug concentration in the tumor than non-functionalized nanoparticles or the free drug one hour and 24 h after administration. The drug concentration increased by 2.6–3.3 times after one hour and 2.7–6.6 times, respectively, after 24 h.
					After administering cationic BSA-conjugated PEG nanoparticles containing aclarubicin to rats, in vivo, tests indicated an increase in their survival rates.
					Encapsulating the drug in nanoparticles helps to reduce its toxicity.
BSA-based nanoparticles cross-linked with GA containing the encapsulated drug imatinib [521].	Desolvation	80–90		Inhibition of receptors such as c-Kit and PDGFR that are overexpressed in glioblastoma.	The study found that the HSA-based nanoparticles had a high encapsulation efficiency percentage of 98% and a drug loading degree of 6.9%.
					HSA nanoparticles with an encapsulated drug concentration of 40 mg/mL had 90% cytotoxicity on U87MG glioblastoma cells, while the free drug had 55%.
BSA-based nanoparticles labeled with a fluorescent dye [522].	Nanoprecipitation, ultrasonication.	100–200	BSA was conjugated with borneol using the carbodiimide reaction method with EDC and NHS.	Menthol-modified nanoparticles were internalized into cells via a temperature-dependent active mechanism and a caveolae-mediated endocytosis mechanism.	BSA-based nanoparticles modified with menthol had better brain targeting and were more efficient in overcoming the BBB than other BSA-based nanoparticles modified with different ligands, as shown in in vivo imaging tests.
			Ketone carbonyl muscone can be linked to BSA through reductive Borch amination.		The BSA functionalized with borneol resulted in obtaining nanoparticles with increased permeability through the BBB due to improved lipophilicity, increased endocytosis, and reduced expression of proteins associated with TJs.
			Para-mentha-8-thiolone is used as a menthol analog to couple with BSA using the reaction with 2-iminothiolane hydrochloride.		Menthol-modified nanoparticles can be uptaken from the bloodstream and could enter into the pineal gland, a more efficient drug delivery pathway to the brain than the one mediated by the transferrin receptor.
			The T7 peptide was conjugated to BSA using the identical method utilized for the menthol.		
Nanoparticles based on BSA that contain encapsulated doxorubicin [523].	Desolvation	100–200	The surface of BSA-based nanoparticles was modified by grafting mPEG2000 to the free amino groups within the protein. Additionally, lactoferrin was attached to the surface of the nanoparticles through electrostatic bonds.	The accumulation of BSA-based nanoparticles in tumors occurs through the EPR effect and transcytosis, which is mediated by the low-density lipoprotein receptor. Lactoferrin can interact with this receptor to facilitate the transcytosis process.	When the quantities of mPEG2000 and lactoferrin were increased, it resulted in an increase in the size of the nanoparticles while causing a decrease in the zeta potential.
					The study conducted on healthy rats revealed that nanoparticles based on mPEG2000-modified BSA had a longer circulation time in vivo.
					Nanoparticles modified with a high amount of lactoferrin and mPEG2000 showed the strongest cytotoxicity and the highest internalization efficiency on both BCEC and C6 cell lines, improving the dual-targeting effects.
					The biodistribution of doxorubicin encapsulated in different formulations showed that lactoferrin-modified nanoparticles were able to cause a notable accumulation of doxorubicin in brain tissue, particularly 2 h after injection (with a significance level of *p* < 0.05).
Albumin/poly(2-methacryloyloxyethyl phosphorylcholine)-based nanoparticles loaded with TMZ [524].	In situ free radical polymerization method.	8.72–32.67	Albumin nanoparticles with synaptic acid extracted from mustard conjugated on their surface.	Internalizing nanoparticles conjugated with synaptic acid in cells requires energy and causes a temporary disruption of TJ proteins, P-gps, and claudin-5.	The functionalized nanoparticles obtained are biocompatible and are able to overcome the BBB.
					The delivery systems that have been modified with encapsulated drugs effectively induce cell apoptosis at the tumor site. They have also been shown to increase the survival time of mice with glioma.
Nanotheranostic probes based on albumin and catalase (catalase integrated into phototheranostic nanoprobe with biomimetic albumin) [525].	Desolvation	54.14 ± 5.17 × 14.36 ± 1.34		Protein-mediated transport.	The development of a catalase-integrated biomimetic albumin phototheranostic probe used to perform multimodal imaging, amplify phototherapy, and guide surgery for glioma after overcoming the BBB, accumulating in invasive glioma by binding albumin to overexpressed proteins, was reported.
					The nanoprobe could effectively induce local hyperthermia and increase the level of singlet oxygen based on the attenuated hypoxic glioma microenvironment by decomposing endogenous hydrogen peroxide into oxygen to enhance phototherapy.
					Glioma growth is significantly inhibited, survival time is prolonged, tumor hypoxia is attenuated, apoptosis is enhanced, and anti-angiogenesis effects have been demonstrated in several animal models with a low toxicity for normal tissue.

## Supplementary Materials

The following supporting information can be downloaded at https://www.mdpi.com/article/10.3390/polym15193969/s1: Figure S1. Schematic illustration of albumin nanoparticle synthesis using the desolvation/coacervation method. Figure S2. The schematization of the method used for obtaining nanoparticles based on HSA through the reduction and desolvation method [284]. Figure S3. Obtaining nanoparticles via thermally induced aggregation. Figure S4. The schematization of the emulsion technique used for obtaining nanoparticles. Figure S5. Schematic of the method of obtaining nanoparticles via self-assembly. Figure S6. The schematization of the method used for obtaining albumin nanoparticles via spray-drying. Figure S7. Schematic representation of the flow system, including the pumps and the μ-mixer cell used to obtain the albumin nanoparticles [301]. Figure S8. Schematization of NAB technology. Preparation of nanoparticles [310].
