# Supplementary material for: Recent Advancements and Strategies for Overcoming the Blood–Brain Barrier Using Albumin-Based Drug Delivery Systems to Treat Brain Cancer, with a Focus on Glioblastoma
Source: Polymers (Basel). 2023 Oct 2;15(19):3969. doi: 10.3390/polym15193969 (PMC10575401; doi:10.3390/polym15193969)
Supplement: Supplementary file 1 [file polymers-15-03969-s001.zip › polymers-2584848-supplementary.pdf]

Supplementary materials

The article's title: Recent Advancements and Strategies for Overcoming the Blood–Brain Barrier Using Albumin-Based Drug Delivery Systems to Treat Brain Cancer, with a Focus on Glioblastoma

Authors: Camelia-Elena Tincu (Iurciuc), Călin Vasile Andrițoiu, Marcel Popa and Lăcrămioara Ochiuz

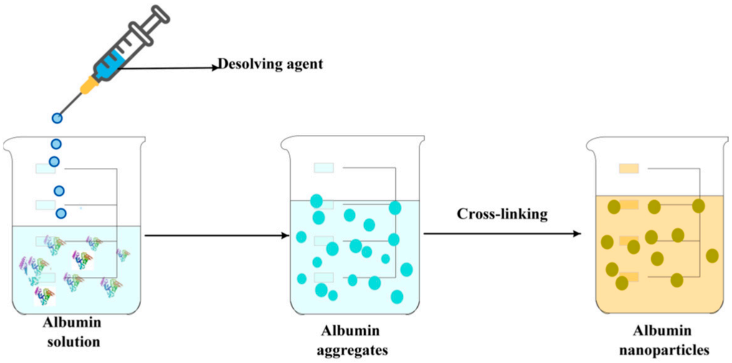

Figure S1. Schematic illustration of the albumin nanoparticles synthesis using the desolvation/coacervation method.

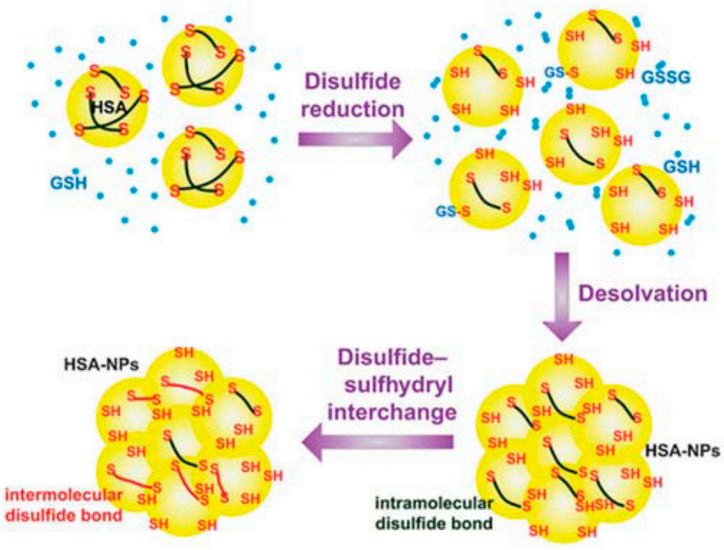

Figure S2. The schematization of the method used for obtaining nanoparticles based on HSA through a reduction and desolvation method [289].

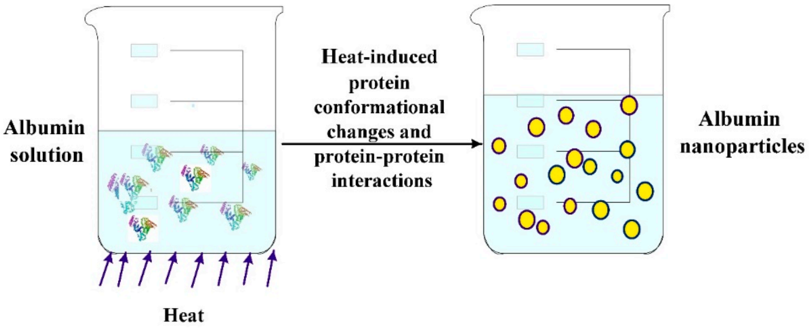

Figure S3. Obtaining nanoparticles by thermally induced aggregation.

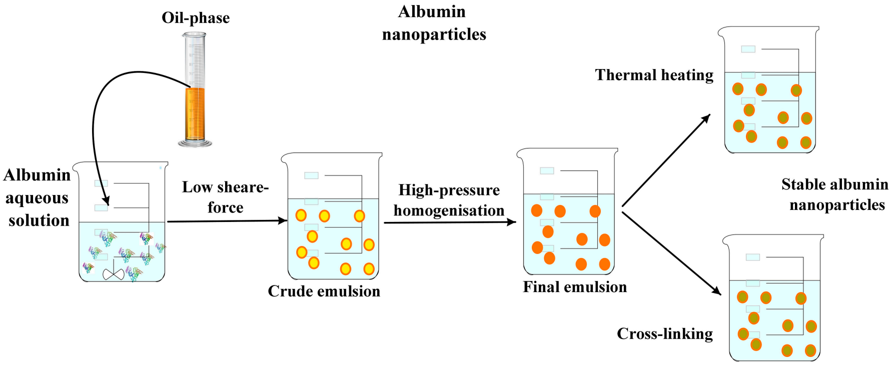

Figure S4. The schematization of the emulsion technique used for obtaining nanoparticles.

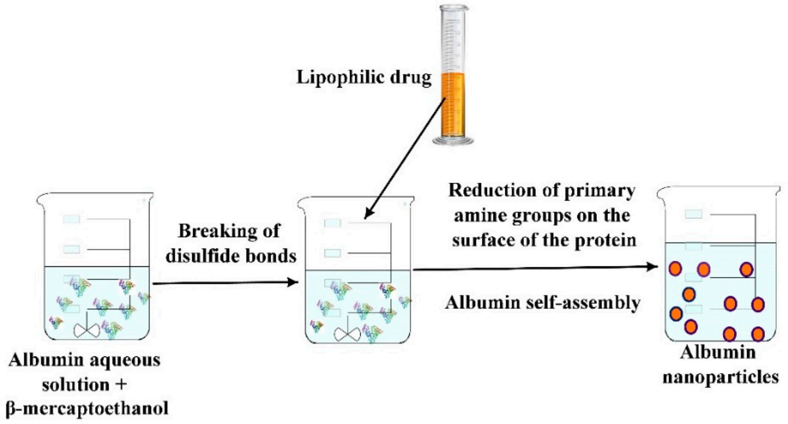

Figure S5. Schematic of the method of obtaining nanoparticles by self-assembly.

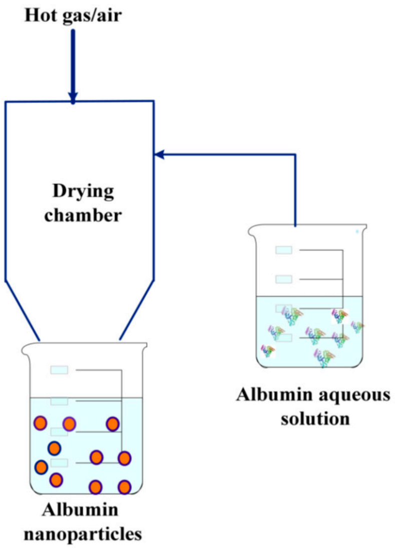

Figure S6. The schematization of the method used for obtaining albumin nanoparticles by spray-drying.

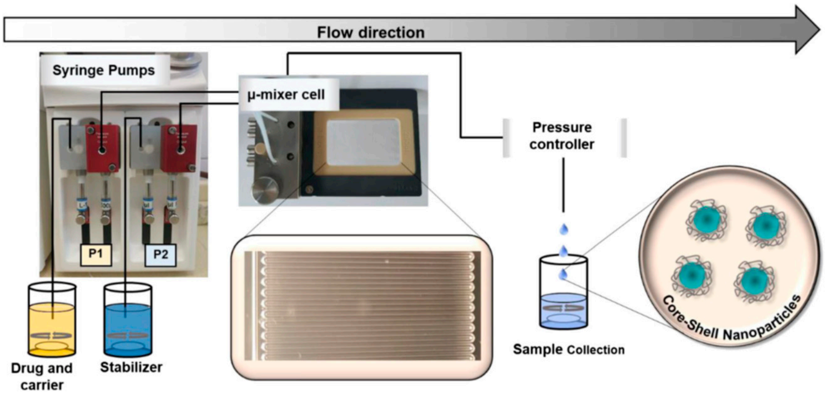

Figure S7. Schematic representation of the flow system, including the pumps and the μ-mixer cell used to obtain the albumin nanoparticles [307].

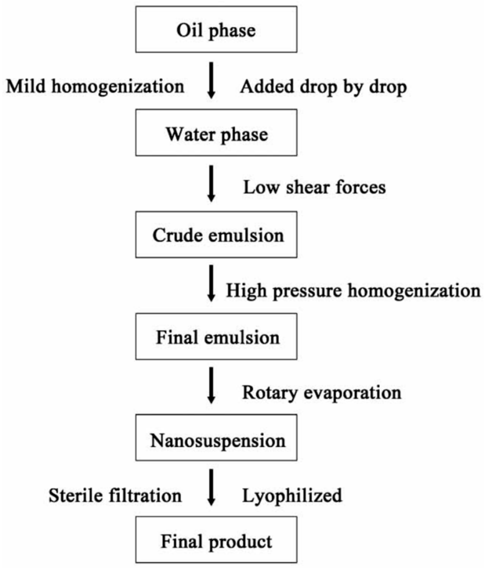

Figure S8. Schematization of NAB-tehnology. Preparation of nanoparticles [319].
